# Time-dependent Pax3-mediated chromatin remodeling and cooperation with Six4 and Tead2 specify the skeletal myogenic lineage in developing mesoderm

**DOI:** 10.1371/journal.pbio.3000153

**Published:** 2019-02-26

**Authors:** Alessandro Magli, June Baik, Lauren J. Mills, Il-Youp Kwak, Bridget S. Dillon, Ricardo Mondragon Gonzalez, David A. Stafford, Scott A. Swanson, Ron Stewart, James A. Thomson, Daniel J. Garry, Brian D. Dynlacht, Rita C. R. Perlingeiro

**Affiliations:** 1 Department of Medicine, Lillehei Heart Institute, University of Minnesota, Minneapolis, Minnesota, United States of America; 2 Department of Pediatrics, University of Minnesota, Minneapolis, Minnesota, United States of America; 3 Department of Molecular and Cell Biology, University of California, Berkeley, California, United States of America; 4 Morgridge Institute for Research, Madison, Wisconsin, United States of America; 5 Department of Pathology, New York University Cancer Institute, New York University School of Medicine, New York, New York, United States of America; Biomedical Center Munich, GERMANY

## Abstract

The transcriptional mechanisms driving lineage specification during development are still largely unknown, as the interplay of multiple transcription factors makes it difficult to dissect these molecular events. Using a cell-based differentiation platform to probe transcription function, we investigated the role of the key paraxial mesoderm and skeletal myogenic commitment factors—mesogenin 1 (Msgn1), T-box 6 (Tbx6), forkhead box C1 (Foxc1), paired box 3 (Pax3), Paraxis, mesenchyme homeobox 1 (Meox1), sine oculis-related homeobox 1 (Six1), and myogenic factor 5 (Myf5)—in paraxial mesoderm and skeletal myogenesis. From this study, we define a genetic hierarchy, with Pax3 emerging as the gatekeeper between the presomitic mesoderm and the myogenic lineage. By assaying chromatin accessibility, genomic binding and transcription profiling in mesodermal cells from mouse and human Pax3-induced embryonic stem cells and Pax3-null embryonic day (E)9.5 mouse embryos, we identified conserved Pax3 functions in the activation of the skeletal myogenic lineage through modulation of Hedgehog, Notch, and bone morphogenetic protein (BMP) signaling pathways. In addition, we demonstrate that Pax3 molecular function involves chromatin remodeling of its bound elements through an increase in chromatin accessibility and cooperation with sine oculis-related homeobox 4 (Six4) and TEA domain family member 2 (Tead2) factors. To our knowledge, these data provide the first integrated analysis of Pax3 function, demonstrating its ability to remodel chromatin in mesodermal cells from developing embryos and proving a mechanistic footing for the transcriptional hierarchy driving myogenesis.

## Abstract 2

Lineage specification during embryonic development requires activation of transcriptional programs to define cellular functions within each tissue. Here, transcriptomic, chromatin accessibility and genomic occupancy studies offer novel insights into the molecular mechanism behind Pax3-mediated specification of the skeletal myogenic lineage.

## Author summary

Lineage specification during embryonic development relies on the activation of specific transcriptional programs required for defining cellular functions within each tissue. Transcription factors (TFs) play a critical part in gene activation/repression by promoting the formation of regulatory hubs at the elements associated with their target genes. Through hierarchical expression of TFs, cells within the developing embryo gradually shape their genomic landscape and ultimately restrict their differentiation potential. Here, we dissect the complex network of TFs involved in the specification of mesoderm toward the skeletal myogenic lineage by generating embryonic stem cell lines in which TF expression is controlled by a doxycycline-dependent on–off system. Using a combination of cell differentiation and genomic assays, we defined the TF hierarchy “Msgn1→Pax3→Myf5,” in which paired box 3 (Pax3) emerges as the key determinant for the specification of the myogenic fate. We show that Pax3 occupies several sites proximal to myogenic genes and that binding to these elements is associated with increase in chromatin accessibility, a hallmark of active regulatory elements. Although the ability of Pax3 to modulate chromatin accessibility is comparable between myogenic and nonmyogenic cells, Pax3 genomic binding and motif preferences are cell-dependent. In addition, we identify that cooperation with sine oculis-related homeobox 4 (Six4) and TEA domain family member 2 (Tead2) is instrumental for the robust Pax3-mediated myogenic commitment, thus indicating that combination of TFs and chromatin remodeling are both required for lineage commitment.

## Introduction

Embryonic development is characterized by a highly regulated cascade of cell fate choices, involving the concerted action of signaling pathways and transcriptional responses, which ultimately results in the specification of progenitors for a specific organ or tissue. The definition of the transcriptional regulatory mechanisms that govern this process is essential for an in-depth understanding of embryogenesis and therefore associated congenital diseases. Transcription factors (TFs) modulate the expression of lineage-specific genes by binding genomic elements, located either in proximity to transcription start sites (TSSs) or at distal intra- and intergenic regions, referred to as enhancers [1]. The enhancer landscape is instrumental for lineage-specific transcription, and chromatin remodeling at these sites is associated with transcriptional changes [2]. Although the nucleosome can represent a physical barrier for TF binding to the DNA, a specific class of TFs known as “pioneers” can overcome this inhibition by engaging their targets on nucleosomal DNA [3]. Since lineage specification often involves multiple classes of TFs, the interplay between pioneering activity and formation of macromolecular complexes that initiate transcription is critical for the successful activation of the differentiation program.

The skeletal myogenic lineage is specified within the somites, epithelial aggregates of paraxial mesodermal progenitors arising from segmentation of the presomitic mesoderm [4]. As the embryo develops, newly formed somites receive cues from the surrounding cells, which result in the specification of the dermomyotome and sclerotome (reviewed by [5]). Skeletal muscle progenitor specification occurs within the central domain of the dermomyotome, from which cells delaminate from the epaxial and hypaxial regions to give rise to trunk and limb muscles, respectively [5]. Although expression of the muscle regulatory factors (MRFs; Myf5, myogenic differentiation protein [MyoD], Mrf4, and myogenin [MyoG]) formally defines the activation of the myogenic program, different sets of TFs act directly or indirectly prior to MRF expression to first specify the somitic mesoderm and subsequently the myogenic fate [5]. *Msgn1* and *Tbx6* play a fundamental role in the differentiation of neuromesodermal progenitors into presomitic mesoderm [6,7]. *Foxc1/2* TFs are expressed in both presomitic mesoderm and formed somites and are necessary for somitogenesis, as evidenced by the lack of somites in *Foxc1*:*Foxc2* compound null embryos [8]. The paired-domain TF *Pax3* is expressed in forming somites and then becomes restricted to the central domain of the dermomyotome, where it is essential for the commitment, survival, proliferation, and migration of myogenic progenitors [9,10]. *Paraxis* (also called *Tcf15*) belongs to the basic helix-loop-helix (bHLH) family of TFs, and it is involved in somite epithelialization [11]. Similarly, *Meox1/2* regulate epithelialization and somite rostrocaudal patterning, with double-knockout (KO) animals displaying severe defects in the differentiation of somite derivatives [12]. *Six1/4* are expressed in both somites and developing muscles, regulating myogenesis at multiple levels, as demonstrated by the muscle hypoplasia observed in *Six1*-null mice and the severe impairment of hypaxial musculature in *Six1*:*Six4* compound mutant embryos [13–15].

Multiple lines of evidence suggest that the hierarchical expression of these TFs is required for the proper activation of the skeletal myogenic lineage. Msgn1 regulates the expression of several paraxial mesoderm genes, including *Pdgfra*, *Foxc1*, and *Snai1* [16]. *Pax3* expression is impaired in both *Meox1*:*Meox2* and *Six1*:*Six4* compound null embryos [12,13]. The myogenic determinant *Myf5* is a well-known critical downstream target gene of paired box 3 (Pax3), which binds at least two well-characterized enhancers located −57 kb and −111 kb from the *Myf5* TSS [17–19]. Similarly, sine oculis-related homeobox 1/4 (Six1/4) and mesenchyme homeobox 2 (Meox2) participate in *Myf5* regulation by binding to the enhancers occupied by Pax3 [20,21]. Nevertheless, key aspects of the molecular mechanism behind the function of these TFs have not been examined, because of the lack of a scalable cellular model able to mimic the specification of the dermomyotome during development. To overcome this limitation, our laboratory has developed a pluripotent stem cell–based model system to recapitulate mouse and human embryonic myogenesis in the dish (reviewed by [22]).

Here, we provide a comprehensive analysis of the mechanisms underlying specification of the skeletal myogenic lineage from mesodermal cells. Using a doxycycline (dox)-inducible mouse embryonic stem (ES) cell–based system, we screened the function of known TFs expressed during the transition from mesoderm to myotome. This revealed a genetic hierarchy involving Msgn1→Pax3→Myf5, in which Pax3 represents the main determinant for the progression of paraxial mesoderm precursors toward the skeletal myogenic lineage. We identify the Pax3 target loci and show that in both differentiating ES cells and developing mouse embryos, Pax3 specifies paraxial mesoderm toward skeletal muscle through chromatin remodeling of these elements. We show that Pax3-induced chromatin remodeling is cell-type independent and that Pax3 myogenic activity requires genetic interaction with Six4 and TEA domain family member 2 (Tead2) TFs.

## Results

### Robust activation of the myogenic program occurs only upon Pax3 and Myf5 expression

**Fig 1:**
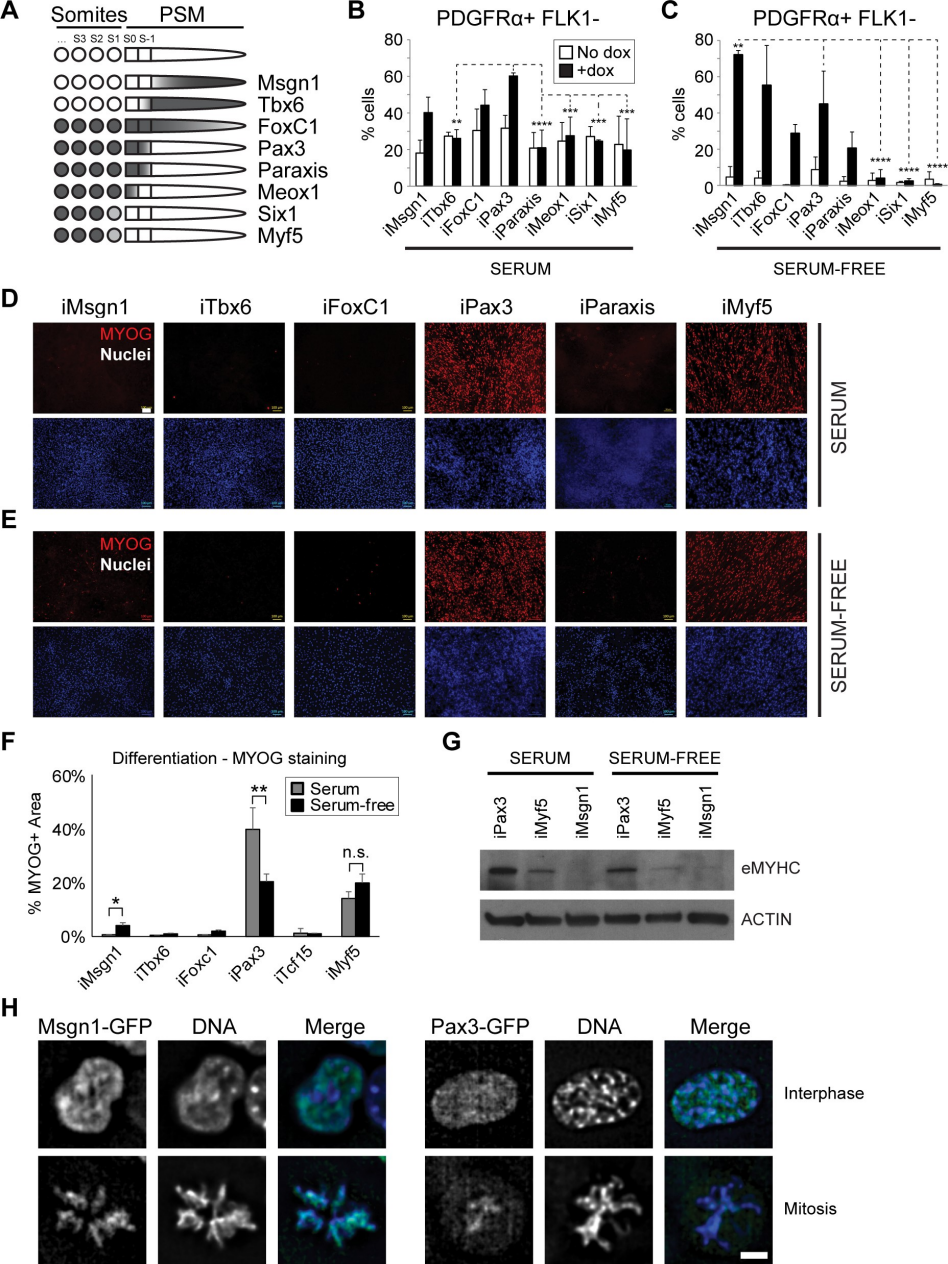
Distinct functions of Msgn1, Pax3, and Myf5 during mesoderm specification. (A) Schematic representation of the developmental expression pattern of the TFs analyzed in this study based on published literature [5–15]. (B-C) Graphs show the percentage of PDGFRα+FLK1− cells, measured by FACS, in day 5 EB cultures from A2lox-Pax3, A2lox-FoxC1, A2lox-Meox1, A2lox-Msgn1, A2lox-Myf5, A2lox-Paraxis, and A2lox-Six1 ES cell lines, using both serum (B) and serum-free (C) differentiation protocols. Mean + SD is shown from at least 3 biological replicates. **p < 0.01, ***p < 0.001, ****p < 0.0001. Differences are relative to the Pax3+dox group. (D-E) Immunofluorescence staining for MyoG in serum (D) and serum-free (E) day 10 cultures following 24-hour dox withdrawal. Images are representative of 3 biological replicates. MYOG (red); nuclei (blue). Bar: 100 μm. (F) Quantification of the MYOG+ area of the immunostaining images shown in Fig 1D and 1E. Graph represents mean + SD from at least 3 independent experiments. *p < 0.05, **p < 0.01. (G) Western blot of day 10 cultures (same as panel D-E) from serum- and serum-free differentiation of A2lox-Pax3, A2lox-Myf5, and A2lox-Msgn1 ES cell lines. eMYHC. ACTIN. (H) Live cell imaging of Msgn1-GFP and Pax3-GFP fusion proteins using wide-field microscopy followed by image deconvolution. Images are representative of 3 biological replicates, and the results were similar between serum- and serum-free differentiation. DNA was visualized using Hoechst 33342. Bar: 5 μm. Numerical values are available in S1 Data. dox, doxycycline; EB, embryoid body; eMYHC, embryonic myosin heavy chain; ES, embryonic stem; FACS, fluorescence-activated cell sorting; FoxC1, forkhead box C1; Meox1, mesenchyme homeobox 1; Msgn1, mesogenin 1; Myf5, myogenic factor 5; MyoG, myogenin; Pax3, paired box 3; PSM, presomitic mesoderm; Six1, sine oculis-related homeobox 1; Tbx6, T-box 6; TF, transcription factor.

To study the role of TFs involved in the specification of the presomitic mesoderm toward the myogenic lineage (Fig 1A), we used an inducible cassette exchange system [23], which allowed for the generation of dox-inducible mouse ES cell lines expressing Msgn1, Tbx6, FoxC1, Pax3, Paraxis, Meox1, Six1, or Myf5 (S1A Fig). Following differentiation into embryoid bodies (EBs), we assessed the ability of these TFs to promote mesoderm lineage specification using fluorescence-activated cell sorting (FACS). As we demonstrated previously, EB-derived PDGFRα+FLK1− cells represent paraxial mesodermal precursors that, upon Pax3 induction, undergo skeletal muscle commitment and ultimately generate a population of proliferating myogenic progenitors endowed with in vivo regenerative potential [24,25]. Besides Pax3, only FoxC1 and Msgn1 were able to induce the PDGFRα+FLK1− fraction, whereas Meox1, Myf5, Paraxis, Six1, and Tbx6 were unable to promote paraxial mesoderm in the presence of serum (Fig 1B and S1B Fig). To better visualize the mesoderm-promoting activity of these factors, the same analysis was performed using a serum-free differentiation protocol, conditions under which mesoderm does not form [26]. As expected, the frequency of PDGFRα+ and FLK1+ cells in noninduced cultures was drastically reduced in the absence of serum. Upon dox induction, significant up-regulation of the PDGFRα+FLK1− fraction was observed not only for Pax3, Msgn1, and FoxC1 but also for Tbx6 and Paraxis (Fig 1C and S1B Fig). The ability to induce platelet-derived growth factor alpha (PDGFRα) in serum-free conditions correlated with the spatial location of these TFs along the anterior–posterior (AP) axis, with the highest PDGFRα induction observed for the more posterior TFs (Msgn1, Tbx6, Foxc1, and Pax3). These data were further confirmed by gene expression analysis of somitic/myogenic markers, as shown by up-regulation of *Meox1*, *Pax3*, and *Paraxis* following Msgn1 and Tbx6 induction in serum-free differentiation (S1C Fig). Since PDGFRα marks both presomitic mesoderm and somites, we next examined whether the PDGFRα+FLK1− cell fractions isolated from serum- and serum-free cultures were endowed with skeletal myogenic potential. Upon sorting, monolayer cultures from the dox-treated inducible-TF cell lines were assayed for the expression of MYOG. Pax3 and Myf5 induction robustly activated the myogenic program in both differentiation conditions (Fig 1D and 1F, S1D Fig), a result in agreement with their function in the myogenic hierarchy. Among the other TFs tested, only Msgn1 displayed a partial capability to induce MYOG in serum-free cultures (Fig 1D, 1E, 1F and 1G). These results imply that Msgn1 functions are affected by serum components and that this TF is not sufficient for the transition of PDGFRα+ cells into the myogenic lineage. Although Msgn1 has been reported to regulate paraxial mesoderm commitment [16], this study did not address whether the resulting cells were capable of cell-autonomous differentiation toward one of the other paraxial mesoderm derivatives.

Since transcriptional activity of Six TFs requires expression of the eyes absent (Eya) proteins, we verified whether these important cofactors are expressed. As shown in S1E Fig, our transcriptomic analyses (described later) indicate these important coactivators are expressed in PDGFRα+ cells, thus suggesting that lack of myogenic activity observed upon Six1 induction is not affected by Eya gene expression (S1D Fig). Nonetheless, we cannot exclude that other TFs tested in this study may not be functional because of the absence of important cofactors.

Mitotic bookmarking has been proposed to play an important role in cell specification during development because of the faster reactivation of mitotically marked genes [27,28]. We reasoned that Msgn1 and Pax3 may differ in their ability to bind chromatin during mitosis and ultimately induce the expression of key genes important for the specification of the myogenic lineage. To test this hypothesis, we generated dox-inducible Msgn1-GFP and Pax3-GFP ES cell lines to visualize these proteins without the use of crosslinking agents, which have been reported to affect mitotic binding [29]. Characterization of these lines demonstrated that C-terminal green fluorescent protein (GFP) fusion enabled the detection of GFP fusion proteins by live cell imaging without affecting Msgn1 and Pax3 function (S1F and S1G Fig). Although we observed clear localization of Msgn1-GFP to mitotic chromosomes, this was not the case for Pax3-GFP, which was barely detected at condensed chromatin (Fig 1H and S1G Fig). Based on these data, we conclude that the inability of Msgn1 to specify the myogenic lineage is not related to mitotic bookmarking but is rather due to its target selectivity.

### Pax3: A gatekeeper of the myogenic lineage

**Fig 2:**
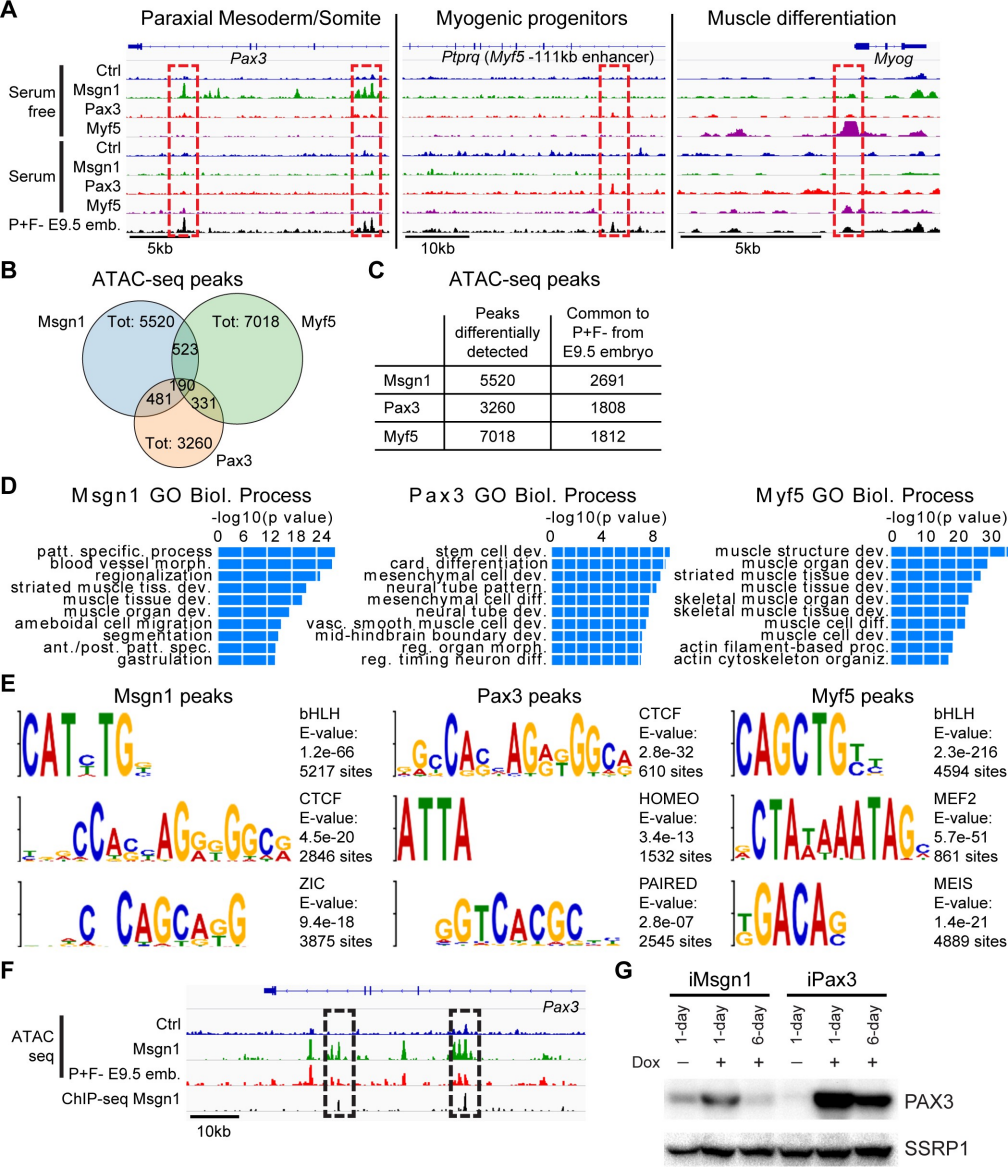
Pax3 as a gatekeeper of the myogenic lineage. (A) Representative IGV tracks for genes associated with paraxial mesoderm/somite formation, myogenic progenitor specification (“spec.”), and muscle differentiation (“diff.”) and comparison with PDGFRα+FLK1− cells isolated from E9.5 mouse embryos (“P+F- E9.5 emb.”). (B) Number of ATAC-seq peaks from serum-free differentiation identified upon induction of Msgn1, Pax3, and Myf5. Venn diagram indicates the overlap between these 3 datasets. (C) Number of peaks from serum-free differentiation overlapping to ATAC-seq peaks detected in PDGFRα+FLK1− cells isolated from E9.5 mouse embryos. (D) Functional classification of Msgn1-, Pax3-, and Myf5-induced accessible peaks from serum-free differentiation using GREAT. Complete annotation data are reported in S2 Table. (E) Selected motifs associated with Msgn1-, Pax3-, and Myf5-induced peaks in serum-free differentiation. (F) IGV snapshot of the Pax3 locus. Changes in chromatin accessibility induced by Msgn1 in serum-free differentiation overlap to peaks identified in the Msgn1 ChIP-seq (dashed black squares). (G) Western blot analysis of PAX3 expression in Msgn1- and Pax3-induced cultures following 1-day and 6-day dox treatment. SSRP1 was used as loading control (“Ctrl”). Numerical values are available in S1 Data. ant./post., anterior–posterior; ATAC-seq, assay for transposase-accessible chromatin sequencing; bHLH, basic helix-loop-helix; Biol., biological; card., cardiac; ChIP-seq, chromatin immunoprecipitation sequencing; CTCF, CCCTC-binding factor; dev., development; dox, doxycycline; E, embryonic day; GO, gene ontology; IGV, Integrative Genomics Viewer; MEF2, myocyte enhancer factor 2; morph., morphology; Msgn1, mesogenin 1; Myf5, myogenic factor 5; organiz., organization; patt., pattern; Pax3, paired box 3; proc., process; reg., regulation; SSRP1, structure-specific recognition protein; Tot, total; vasc., vascular.

To dissect the differences between Msgn1 and Pax3 at the epigenetic level and provide mechanistic insight into the transition from presomitic mesoderm to somite-myotome, we employed the assay for transposase-accessible chromatin sequencing (ATAC-seq) [30] to map genome-wide changes in chromatin accessibility. For this analysis, we focused on day 4 cells from serum and serum-free differentiation EB cultures upon induction of Msgn1, Pax3, or Myf5. PDGFRα+FLK1− cells isolated from the trunk region of embryonic day (E)9.5 mouse embryos were used as reference (Fig 2A, “P+F− E9.5 emb.”). Compared to day 4 noninduced EBs, all TFs provoked changes in chromatin accessibility at genomic regions associated with expected or known targets: *Pax3* and *Paraxis* in the case of Msgn1, the *Myf5* −111 kb enhancer [18] and *Met* for Pax3, and the *Myogenin* promoter [31,32] and *muscle creatine kinase* (*Ckm*) for Myf5 (Fig 2A and S2A Fig). In agreement with our data (Fig 1 and S1 Fig), we only observed changes at genes involved in somitogenesis when Msgn1 was induced in serum-free conditions (Fig 2A and S2A Fig). Globally, we detected 5,520, 3,260, and 7,018 new accessible regions after induction of Msgn1, Pax3, and Myf5 in serum-free conditions, respectively (Fig 2B and S1 Table). A total of 2,691/5,520 (49%) and 1,808/3,260 (55%) of the ATAC-seq peaks induced by Msgn1 and Pax3, respectively, overlapped with accessible peaks detected in E9.5 embryos (Fig 2C). In the case of Myf5, we observed only 1,812/7,018 (26%) overlapping peaks between day 4 EBs and E9.5 embryos, likely because the full expression of this gene occurs at later developmental stages (Myf5 protein is detectable from E10.5).

Using the annotation tool GREAT [33], we annotated the differentially accessible genomic regions for each TF (Fig 2D, S2B Fig, and S2 Table). Msgn1- and Pax3-induced accessible loci were associated with genes involved in various mesodermal cell categories, including segmentation, muscle, and mesenchymal cell development (Fig 2D and S2B Fig). In contrast, Myf5 induction increased accessibility at loci involved in later stages of skeletal muscle differentiation, such as *Myog* and *Ckm* (Fig 2D), which is in agreement with the ability of the myogenic determinants to activate the skeletal muscle program in multiple cell types, including nonmesodermal cells [34]. This finding indicates that the ability of Myf5 to induce myogenesis also in PDGFRα-negative cells (S1B Fig) involves a mechanism that is distinct from that of Pax3, which occurs only in mesodermal cells (further discussed in this manuscript). In addition, although we observed increased chromatin accessibility at the *Myf5* enhancer following 1-day Pax3 induction, Myf5 mRNA expression increased robustly during the next 5 days (S1E Fig), which recapitulates the pattern of Myf5 expression in developing embryos [35,36]. This also corresponded to increased chromatin accessibility at the *Myog* locus (S2C Fig). Based on these data, we believe that Myf5 downstream targets (such as Myog) are activated only upon robust Myf5 induction, which occurs through sustained Pax3 expression.

To demonstrate these three TFs regulate different subsets of loci, we compared the chromatin accessibility profiles of Pax3-, Msgn1-, and Myf5-induced EB cells to E9.5 embryos (S2B Fig and S2 Table). In agreement with the pairwise comparison of Fig 2B, combined analysis of these ATAC-seq data shows minimal overlap of the loci characterized by increased chromatin accessibility upon Msgn1, Pax3, and Myf5 induction (S2B Fig), which is further confirmed by gene ontology (GO) analysis of the loci associated to each cluster (S2 Table). Motif analysis on the accessible peaks induced by each TF (total number from Fig 2B) confirmed the enrichment for binding sites associated with bHLH factors, such as Msgn1 and Myf5, and paired domain, as in the case of Pax3 (Fig 2E and 2F). We observed enrichment of the CCCTC-binding factor (CTCF) motif in both Msgn1 and Pax3 peaks, thus suggesting that lineage specification transitions induced by these TFs may be associated with changes in chromosome architecture. This was not the case for the Myf5 peaks enriched for myocyte enhancer factor 2 (MEF2) and MEIS motifs, which have been shown to cooperate with the myogenic bHLH proteins (Fig 2E) [37,38].

An interesting hypothesis that can be drawn from these ATAC-seq data is the potential direct involvement of Msgn1 in *Pax3* regulation (Fig 2A and S1B Fig). To investigate this, we compared the chromatin accessibility profile at the Pax3 locus with published Msgn1 chromatin immunoprecipitation sequencing (ChIP-seq) data [16]. As shown in Fig 2F, Msgn1 binding is detected at intronic elements of the Pax3 gene, and both peaks correspond to ATAC-seq peaks found at the Msgn1-inducible EBs and E9.5 embryos. Western blot and ChIP analyses confirmed the increase in PAX3 protein expression following Msgn1 dox-dependent induction in serum-free cultures (Fig 2G) and Msgn1 binding at the Pax3 locus (S2G Fig). Nonetheless, Msgn1-induced PAX3 expression was found to be much lower than that obtained following Pax3 induction, and sustained Msgn1 expression was not sufficient for maintaining Pax3 expression over time (Fig 2G and S2H Fig). These observations may account for Msgn1 incapability to robustly induce myogenesis (Fig 1E and 1F) and suggest the requirement of an additional factor for maintaining PAX3 expression in the trunk somites.

Altogether, these data demonstrate the sequential activation of the genes involved in skeletal myogenic lineage specification from presomitic mesoderm, which follows the hierarchy Msgn1→Pax3→Myf5. Pax3 plays a central role in this process by representing the earliest upstream regulator for the robust specification of the myogenic lineage from paraxial mesoderm (Fig 1D and 1F, S1C and S1D Fig).

### Conserved role of Pax3 in the regulation of somitic/myogenic gene expression

**Fig 3:**
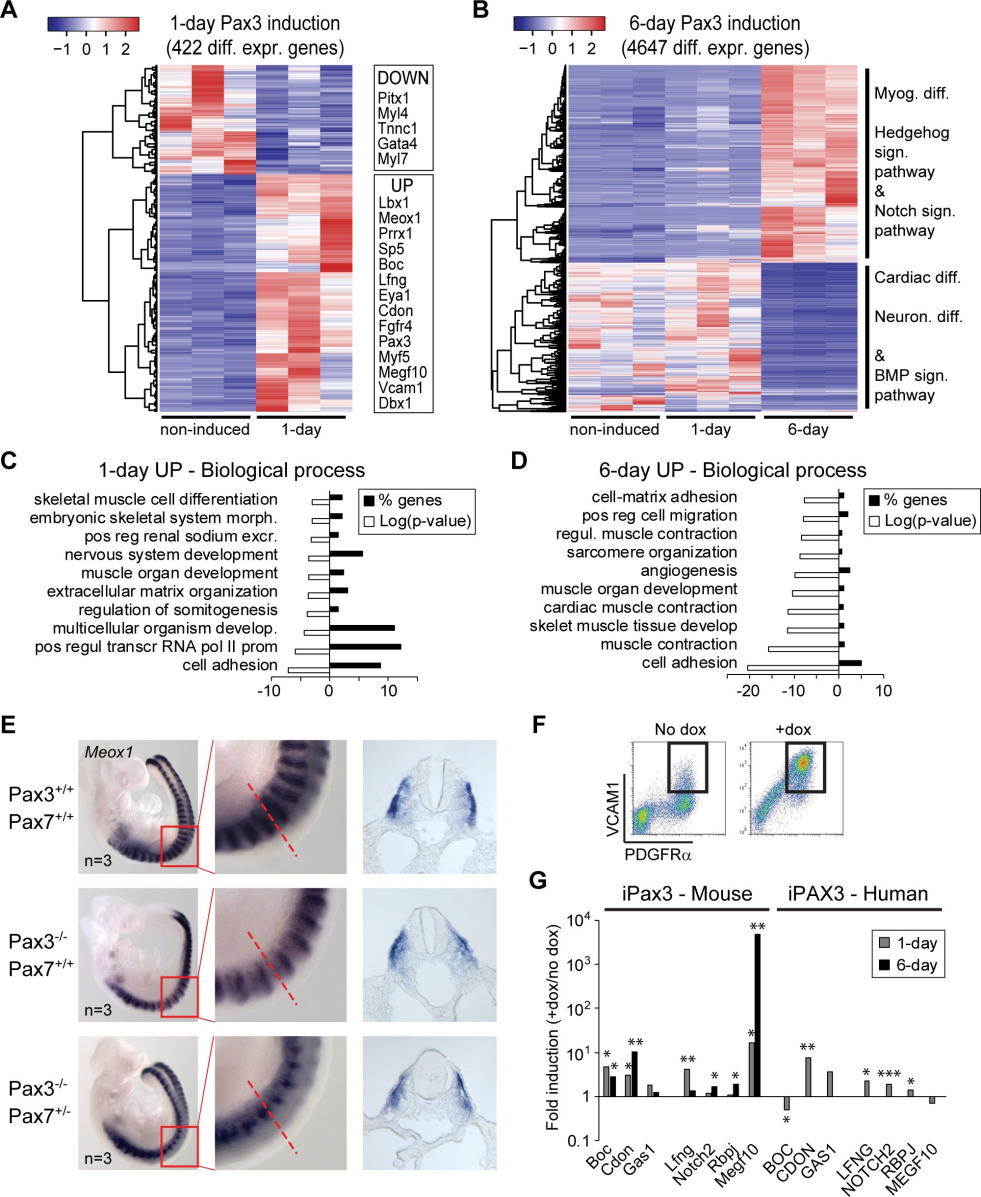
Conserved role of Pax3 in the regulation (“regul.”) of somitic/myogenic gene expression. (A-B) Heatmap of genes up-regulated upon 1-day and 6-day Pax3 induction. Changes are relative to noninduced Pax3 cells and were obtained by the analysis of 3 independent biological replicates. Selected genes and biological processes affected by Pax3 are indicated on the right side of the heatmaps. (C-D) GO analysis of up-regulated Pax3 genes: 1-day and 6-day samples using DAVID. (E) Analysis of Meox1 expression in the somites of WT and Pax3-null;Pax7-het E9.5 embryos using ISH. Red square represents the zoomed region (middle panel). Dashed red line indicates the section displayed in the right panel. (F) Representative FACS analysis of 2-day Pax3-induced EB cultures shows up-regulation of VCAM1 (y-axis). PDGFRα expression is shown on the x*-*axis. (G) Comparison of transcriptional changes for selected genes associated with Hedgehog and Notch signaling (“sign.”) pathways upon Pax3 expression in differentiating mouse and human ES cells. Data were extracted from RNA-seq analysis of 1-day and 6-day Pax3-induced (+dox) and noninduced (no dox) cultures. Bars represent fold induction (+dox/no dox) of each sample’s mean. *p < 0.05, **p < 0.01, ***p < 0.001. Numerical values are available in S1 Data. BMP, bone morphogenetic protein; develop., development; diff., differentiation; diff. expr., differentially expressed; dox, doxycycline; E, embryonic day; EB, embryoid body; excr., excretion; FACS, fluorescence-activated cell sorting; GO, gene ontology; ISH, in situ hybridization; Meox1, mesenchyme homeobox 1; morph., morphogenesis; Neuron., neuronal; Pax, paired box; PDGFRα, platelet-derived growth factor receptor alpha; pos reg, positive regulation; pos regul transcr RNA pol II prom, positive regulation of transcription from RNA pol II promoter; VCAM1, vascular cell adhesion molecule 1; WT, wild-type.

Although the necessity of Pax3 during muscle development has been elegantly demonstrated using transgenic/KO mouse models [17,18,39,40], the molecular mechanisms associated with Pax3 myogenic activity remain mostly unknown. Given the ability of Pax3 to efficiently drive skeletal myogenesis from mesodermal cells, we set out to decode the molecular mechanisms responsible for its activity in early mesodermal cells (1-day Pax3 induction) and myogenic progenitors (6-day Pax3 induction) using unbiased approaches. Transcriptomic analysis of EB-derived cells identified 422 and 4,647 differentially regulated genes following comparison of noninduced cells to 1-day and 6-day Pax3-induced cells, respectively (Fig 3A and 3B). As expected, and in agreement with the ATAC-seq dataset, this last group included many genes expressed in somites (e.g., *Paraxis*, *Meox1*, *Grem1*), myogenic cells (e.g., *Myf5*, *Met*, *Eya1*), Notch signaling pathway (e.g., *Lfng*, *Jag1*, *Megf10*), and Hedgehog (HH) signaling pathway (*Cdon*, *Boc*, *Gas1*) (Fig 3A). Induction of Pax3 in differentiating EBs was also associated with the repression of the cardiac program and bone morphogenetic protein (BMP) signaling pathway, as evidenced by the down-regulation of *Gata4*, *Nkx2-5*, *Hand1*, *Myh7*, *Bmp2*, and *Bmp4*, among others (Fig 3B) [41]. Biological process classification of 1-day Pax3 up-regulated genes showed enrichment for neurogenesis and mesoderm development, both regulated by Pax3 during embryogenesis (Fig 3C and 3D, S3 Table). However, whereas the levels of myogenic transcripts (e.g., Myf5, Lbx1, and multiple epidermal growth factor–like domains protein 10 [Megf10]) showed a time-dependent increase in expression, neuronal genes were down-regulated during the 1-day-to-6-day transition (S3A Fig). In addition, 8% (23/289) of the genes induced by Pax3 were TFs, thus supporting its role as an early master regulator (S3 Table).

Since Pax3-induced cells displayed up-regulation of somitic markers, we next confirmed that Meox1 expression is impaired in the absence of Pax3 in vivo. As shown in Fig 3E, we analyzed *Pax3*-null E9.5 mouse embryos from an F1 cross and found impaired expression of the somite marker *Meox1* in the dorsoventral domain, normally characterized by Pax3 expression. Selected potential Pax3 targets, including *Ebf3*, *Eya1*, *Megf10*, and *Vcam1*, were validated by gene expression analysis in 6-hour Pax3-induced cells (e.g., S3B Fig). Using FACS, we verified that vascular cell adhesion molecule 1 (Vcam1) is strongly up-regulated by Pax3 (Fig 3F).

Using an analogous approach in differentiating human ES cells, we determined the transcriptional changes induced by PAX3 in human mesoderm. As expected, the ability of PAX3 to induce the myogenic program in differentiating human ES cells is conserved (S3C Fig), and, similar to murine cells, within 1-day of dox-dependent induction (from day 5 cultures of differentiating H9 cells) PAX3 up-regulates genes involved in paraxial mesoderm formation (S3D Fig). Unbiased transcriptomic analysis identified 746 human transcripts subjected to PAX3 regulation (388 up-regulated and 358 down-regulated; S3E Fig). GO classification based on biological process showed that the majority of PAX3-induced transcripts are associated with developmental processes, including mesoderm and muscle development (S3F Fig). Comparison of murine and human datasets revealed that 8% (60/746) and 51% (381/746) of the genes regulated in inducible-PAX3 (iPAX3) human cells are also differentially expressed in 1-day and 6-day iPax3 mouse cells, respectively (S3G Fig and S4 Table). As observed with mouse counterparts, genes associated with BMP, Notch, and HH signaling pathways are also regulated by PAX3 in human cells (Fig 3G and S3H Fig). Altogether, these data identify the conserved function of Pax3 in the specification of the myogenic lineage.

### Pax3 regulates chromatin accessibility during myogenic specification

**Fig 4:**
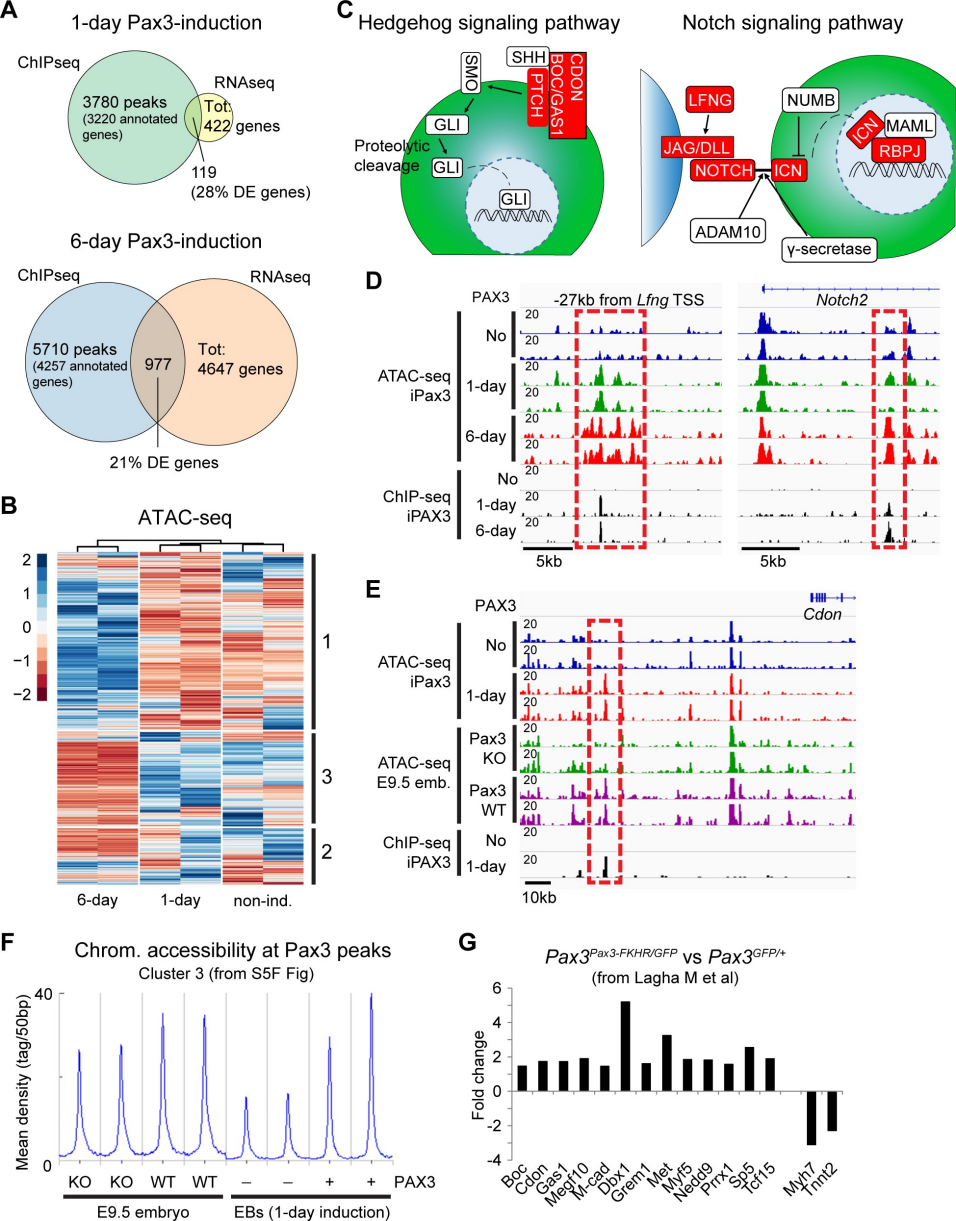
Pax3 induces chromatin remodeling at a subset of bound loci in mesodermal cells. (A) Venn diagrams displaying overlap between DE genes and Pax3-bound loci in 1-day (top) and 6-day (down) samples. Numbers indicate the overlap between the two datasets. (B) Heatmap displaying the changes in chromatin accessibility in PDGFRα+FLK1− cells isolated from noninduced (“non-ind.”), 1-day, and 6-day Pax3-induced cells. Differential accessible loci from 1-day versus noninduced and 6-day versus noninduced were combined in a list of unique peaks and used to generate the differential analysis. Three clusters (indicated on the right side) were identified, and the corresponding coordinates were used for GO analysis. The legend indicates the scaled (*z* score) coverage information for each region. (C) Schematic representation of Hedgehog and Notch signaling pathways. Red blocks indicate genes characterized by increased chromatin accessibility upon Pax3 induction. (D) IGV track displaying chromatin accessibility and Pax3 genomic binding at the *Lfng* and *Notch2* loci in cells isolated from 1-day and 6-day Pax3-induced (+) and noninduced (-) EB cultures. Dashed red squares show Pax3-dependent regulation of chromatin accessibility at the Pax3-bound *Lfng* −25 kb and *Notch2* +10 kb sites. Pax3 binding to these elements are shown by the ChIP-seq tracks. (E) IGV track displaying chromatin accessibility and Pax3 genomic binding at the *Cdon* locus in cells isolated from 1-day Pax3-induced (+) and noninduced (-) EB cultures and Pax3 WT and KO E9.5 embryos (“emb.”). Dashed red square shows Pax3-dependent regulation of chromatin accessibility during embryonic development at the *Cdon* −49 kb site. (F) Distribution of ATAC-seq reads across a subset of 684 Pax3 ChIP-seq peaks generated using *k*-means clustering. Curves show chromatin (“chrom.”) accessibility at Pax3 peaks (±3 kb from peak center) in PDGFRα+FLK1− cells from Pax3 WT and KO embryos and noninduced (-) and 1-day induced (+) EB cells. (G) Selected Pax3 targets are regulated during mouse embryonic development (transcriptomic data published by [43]). Graph represents the expression fold change of selected statistically significant and differentially regulated transcripts from the somites of *Pax3*^*Pax3FKHR/GFP*^ and *Pax3*^*GFP/+*^ embryos. Numerical values are available in S1 Data. ADAM, a disintegrin and metallopeptidase domain 10; ATAC-seq, assay for transposase-accessible chromatin sequencing; BOC, biregional Cdon binding protein; CDON, cell adhesion molecule–related/down-regulated by oncogenes; ChIP-seq, chromatin immunoprecipitation sequencing; DE, differentially expressed; DLL, delta-like canonical Notch ligand; E, embryonic day; EB, embryoid body; GAS1, growth arrest specific 1; GO, gene ontology; ICN, intracellular notch; IGV, Integrative Genomics Viewer; JAG, jagged; KO, knockout; LFNG, lunatic fringe; MAML, Mastermind-like; Pax3, paired box 3; PTCH, patched; RNAseq, RNA sequencing; SHH, Sonic hedgehog; SMO, Smoothened; Tot, total; TSS, transcription start site; WT, wild-type.

To investigate whether Pax3 has a direct function in the regulation of the genes identified in our transcriptomic studies, we analyzed Pax3 genomic occupancy in murine EB-derived cells 1 day and 6 days post dox-mediated induction, using ChIP followed by sequencing (ChIP-seq). As expected, Pax3 binding was detected at genomic loci proximal to many differentially regulated genes (S4A and S4B Fig, S5 Table), even though in multiple cases this occurred many kilobases from the respective TSS. Annotation of 1-day and 6-day ChIP-seq data using PAVIS [42] assigned a peak to 28% (119/422) and 21% (977/4,647) of the 1-day and 6-day RNA sequencing (RNA-seq) differentially expressed genes, respectively (Fig 4A and S6 Table), even though this may represent an underestimation due to localization of the peak in a nearby gene. GO classification of the peaks detected at both time points was consistent with Pax3 function in the regulation of mesoderm (1-day; total 3,780 peaks) and skeletal muscle development (6-day; total 5,710 peaks) (S4C Fig and S2 Table). Moreover, 14% of the 1-day Pax3 peaks were also conserved in human ES-derived counterparts, including elements proximal to the HH coreceptors *CDON* and *GAS1* and the *NOTCH2* receptor (S4D Fig and S5 Table).

Next, we took advantage of chromatin accessibility data to investigate the epigenetic changes occurring during Pax3-mediated specification of the myogenic lineage (Fig 4B and S5A Fig). As exemplified by the *Myod* locus (S5A Fig), comparison of ATAC-seq data between noninduced and 6-day Pax3-induced cells identified an increase in chromatin accessibility at several loci associated with myogenic commitment (S5B Fig and S2 Table). In agreement with our transcriptomic analyses (Fig 3 and S3 Fig), loci identified only in noninduced cells (therefore characterized by decreased chromatin accessibility in 6-day Pax3-induced cells) are enriched for cardiac genes, the BMP signaling pathway, and other nonmyogenic mesodermal biological process categories (S5B Fig and S2 Table). This analysis confirmed the increase in chromatin accessibility at the Pax3-bound elements associated with several genes belonging to HH and Notch signaling pathways (Fig 4D and 4E).

### Chromatin accessibility is impaired in *Pax3*-null embryos

To determine whether chromatin remodeling of these elements requires Pax3 in vivo, we analyzed the changes in chromatin accessibility upon Pax3 loss of function in mouse embryos. For this analysis, we performed ATAC-seq on PDGFRα+FLK1− cells isolated from the trunk region of wild-type (WT) and Pax3-null (KO) E9.5 embryos. Clustering of the ATAC-seq data on the Pax3 ChIP-seq peaks classified 3 groups based on the chromatin accessibility signal (S5D Fig). Whereas groups 1 and 2 showed limited or no changes (S5E Fig), chromatin accessibility of group 3 revealed a Pax3-dependent change in PDGFRα+FLK1− mesodermal cells isolated from mouse embryos as well as from day 4 EBs (Fig 4F). These results confirm that activation of the myogenic program by Pax3 during embryogenesis is associated with chromatin remodeling of its binding sites. For example, we observed a dramatic decrease in accessibility at the *Cdon* −49 kb, *Vcam1* +3 kb, and *Myf5* −111 kb elements (Fig 4E and S5F Fig) and similar results at many other Pax3-bound sites in Pax3-null E9.5 embryos (e.g., *Eya1*, *Jag1*, *Met*, and *Notch2*). In further support of our findings, biregional cell adhesion molecule–related/down-regulated by oncogenes (Cdon) binding protein (Boc) and Cdon transcripts are down-regulated in Pax3-null E9.5 embryos (S5G Fig), and analysis of the transcriptomic changes following expression of dominant positive Pax3-FKHR fusion protein in developing murine somites [43] showed significant up-regulation of several genes identified in our genome-wide studies (Fig 4G).

### Pax3 induces chromatin remodeling independently of the activation of the myogenic program

**Fig 5:**
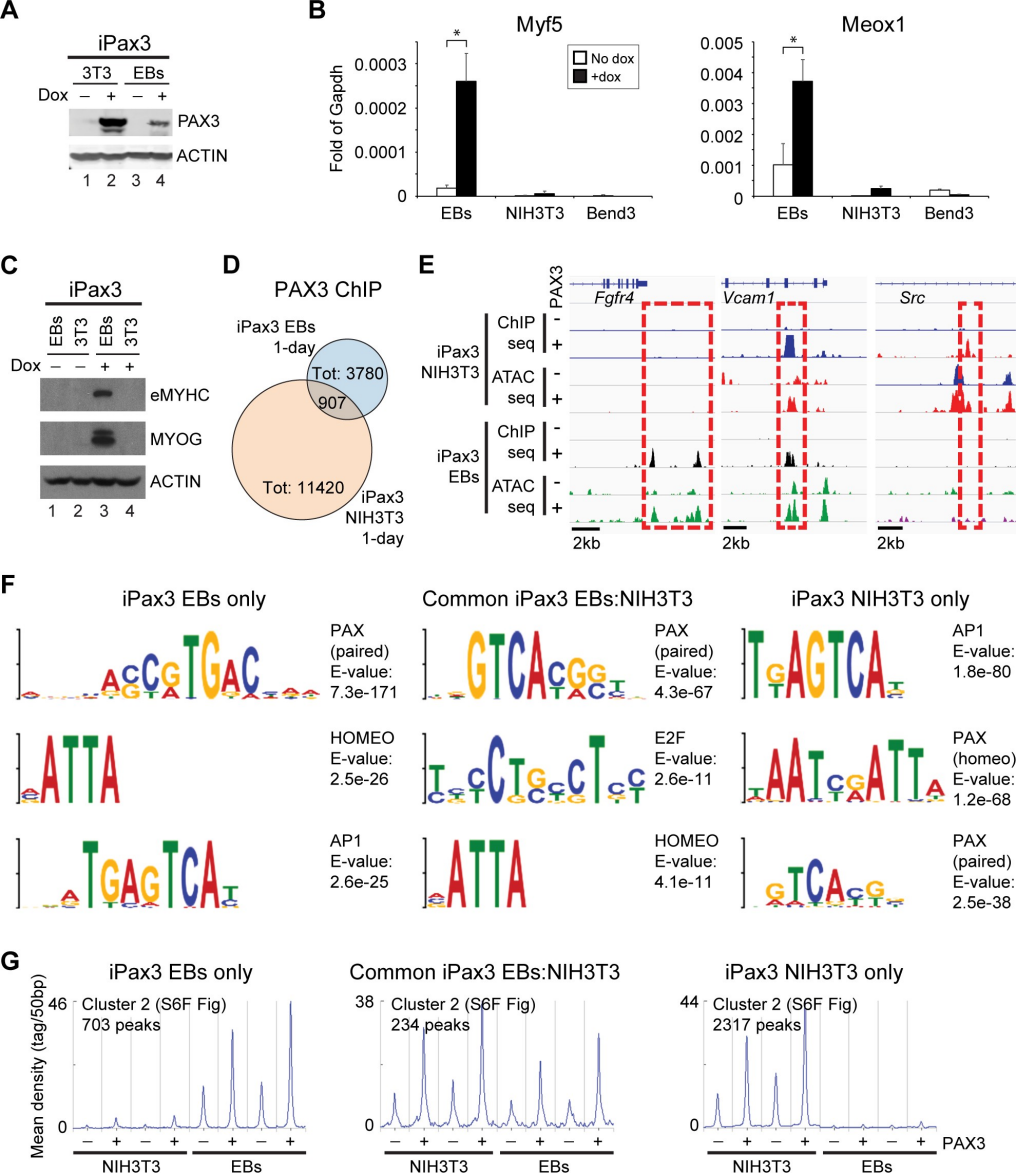
Pax3 does not activate the myogenic program in NIH3T3 fibroblasts and Bend3 endothelial cells. (A) Pax3 induction in EBs and NIH3T3 iPax3 cells was assessed by western blot using antibodies to PAX3 and ACTIN. (B) qRT-PCR analysis for *Myf5* and *Meox1* in Pax3-induced (+dox) and noninduced (no dox) in day 4 EBs, NIH3T3, and Bend3 cells. Graph represents mean + SD from at least 3 independent experiments. *p < 0.05. (C) Cells from Pax3-induced (+dox) and noninduced (no dox) EBs and NIH3T3 cells were analyzed by western blot using antibodies specific for eMYHC, MYOG, and Actin. (D) Venn diagram displaying overlap between Pax3 genomic occupancy in 1-day EBs and NIH3T3 iPax3 cells. EBs-only peaks: 2,873; common EBs/NIH3T3 peaks: 907; NIH3T3-only peaks: 10,513. (E) IGV track displaying genomic occupancy and chromatin accessibility at the *Fgfr4*, *Vcam1*, and *Src* loci in PDGFRα+FLK1− cells isolated from Pax3-induced (+) and noninduced (-) day 4 EBs and Pax3-induced (+) and noninduced (-) NIH3T3 fibroblasts. Dashed red square shows Pax3-dependent regulation of chromatin accessibility. (F) Selected transcription factor motifs enriched at Pax3-bound loci from EBs-only peaks, common EBs/NIH3T3 peaks, and NIH3T3-only peaks. (G) Distribution of ATAC-seq reads across a subset of Pax3 ChIP-seq peaks generated using *k*-means clustering (from S6F Fig). Curves show chromatin accessibility at cluster 2 from EBs-only, common EBs/NIH3T3, and NIH3T3-only Pax3-bound peaks. Datasets are independent biological replicates. Graph represents the ATAC-seq reads overlapping to Pax3 ChIP-seq peaks (Pax3 peak center ± 3 kb). Numerical values are available in S1 Data. AP1, activator protein 1; ATAC-seq, assay for transposase-accessible chromatin sequencing; ChIP-seq, chromatin immunoprecipitation sequencing; dox, doxycycline; EB, embryoid body; eMYHC, embryonic myosin heavy chain; Fgfr4, fibroblast growth factor receptor 4; IGV, Integrative Genomics Viewer; iPax3, inducible-Pax3; MYOG, myogenin; Pax3, paired box 3; qRT-PCR, quantitative reverse transcription PCR; Src, Rous sarcoma oncogene; Tot, total; Vcam1, vascular cell adhesion molecule 1.

Previous studies have demonstrated that Pax3 expression in cell types distinct from PDGFRα+ paraxial mesoderm, such as fibroblasts, does not result in efficient activation of the myogenic program [19,44]. To assess whether this is due to impaired chromatin remodeling of Pax3-bound sites, we introduced the dox-inducible Pax3 system into NIH3T3 fibroblasts (Fig 5A) and Bend3 endothelial cells. The expression of somitic and myogenic genes was minimal or absent upon Pax3 induction in NIH3T3 and Bend3 cells (Fig 5B) and accordingly did not result in the activation of the myogenic program (Fig 5C). Since the PAX family of TFs has been implicated in epigenetic regulation through interaction with the COMPASS histone methyltransferase (HMT) complex and modulation of monomethylated lysine 4 of histone 3 (H3K4me1) [45–48], we evaluated these cells for the deposition of H3K4me1 following Pax3 induction. Following 24 hours of Pax3 induction, H3K4me1 levels in these cells were comparable to EBs at the −111 kb *Myf5* enhancer (S6A Fig) and drastically reduced at the intragenic *Myf5* site (+0.7 kb from TSS). To investigate the global molecular function of Pax3 in fibroblasts, we performed ChIP-seq and ATAC-seq on 1-day induced iPax3 NIH3T3 cells. As shown in Fig 5D, ChIP-seq for Pax3 in NIH3T3 cells identified 11,420 peaks, 907 of which were in common to the 1-day EB dataset. Loci occupied by Pax3 only in EB-derived cells were associated with genes involved in neurogenesis and mesoderm development, such as *Fgfr4* [49] (Fig 5E, S6B Fig and S2 Table). In contrast, peaks identified only in NIH3T3 cells were characterized by enrichment for biological process categories such as regulation of apoptosis and protein phosphorylation, such as *Src* (Fig 5E, S6B Fig and S2 Table). Motif analysis of peaks found solely in EBs or NIH3T3 or common to both revealed interesting differences in the modality of Pax3 binding to these different subsets. Whereas EB-derived Pax3 peaks displayed enrichment for paired-type PAX motifs, NIH3T3-only peaks were characterized by homeodomain- and paired-type PAX motifs (Fig 5F, S6C, S6D and S6E Fig). Accordingly, selectivity between paired- and homeodomain-type motifs has been suggested to play a role in the pioneering activity of Pax7 in melanotrope versus corticotrope differentiation [50], thus supporting Pax3’s ability to bind its targets in a context-dependent manner. Next, using ATAC-seq data from 1-day iPax3 EB and iPax3 NIH3T3 cells, we compared chromatin accessibility at Pax3 binding sites in EBs only, common EBs/NIH3T3, and NIH3T3 only. For all subsets, *k*-means clustering showed 3 groups of loci (S6F Fig). Cluster 1 had the highest accessibility, whereas cluster 3 had the lowest (S6G Fig). Upon dox treatment, we observed subset-specific Pax3-dependent increase in chromatin accessibility in cluster 2, as exemplified by *Fgfr4*, *Vcam1*, and *Src* (Fig 5E and S5G Fig). These sites also displayed distinct basal chromatin accessibility, indicative of cell-specific differences at remodeled sites (Fig 5G). Taken together, these findings support the notion that Pax3 has the ability to induce chromatin remodeling, which is conserved among different cell types, and suggest that activation of the myogenic program depends on the selection of sites bound by this TF.

### Pax3 requires Six4 and Tead2 for efficient induction of skeletal myogenesis

**Fig 6:**
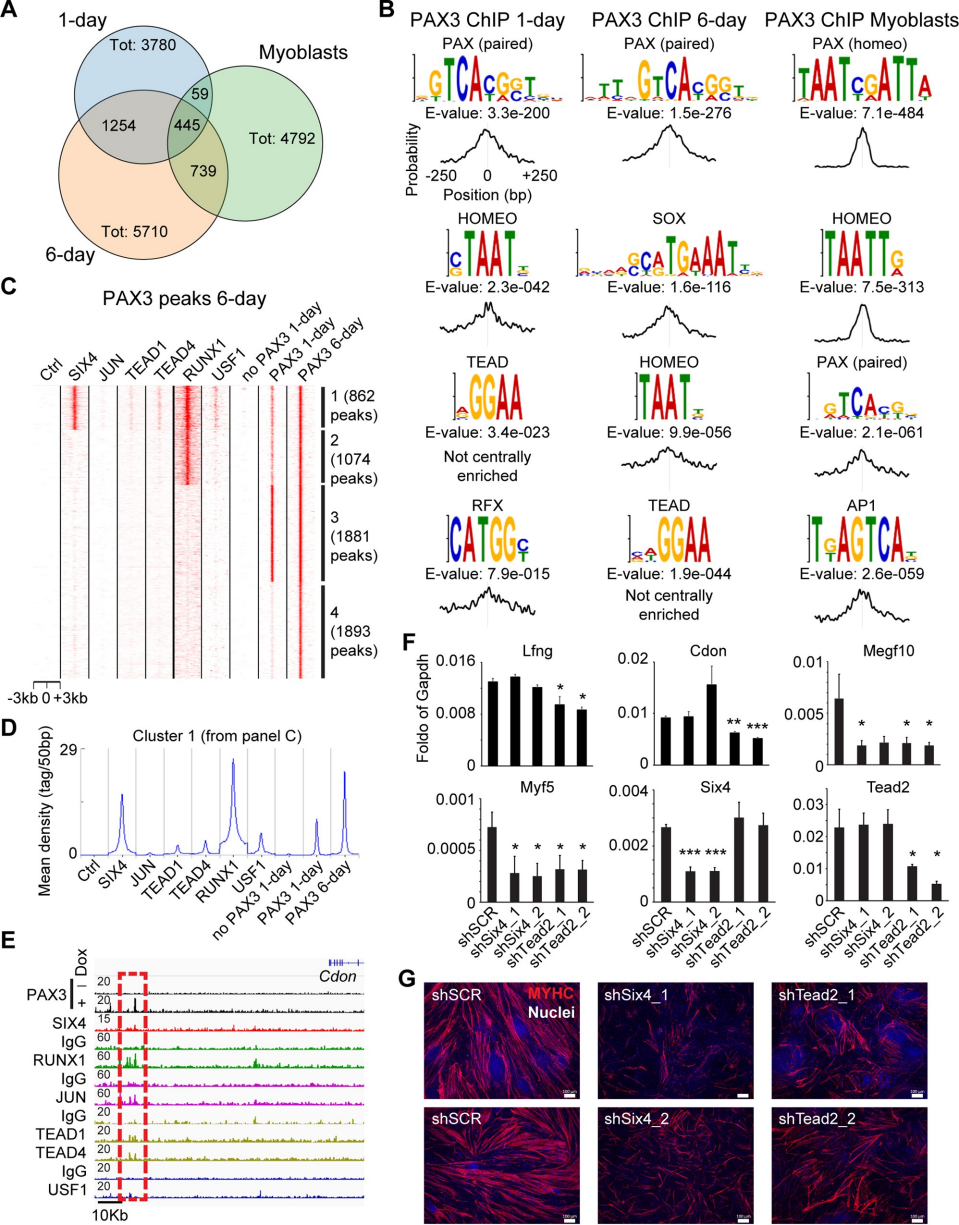
Pax3 cooperates with Six4 and Tead2 to activate the myogenic program. (A) Number of ChIP-seq peaks identified upon PAX3 immunoprecipitation in primary myoblasts, 1-day, and 6-day iPax3-induced cells. Venn diagram indicates the overlap between these 3 datasets. (B) Selected transcription factor motifs enriched at Pax3-bound loci from 1-day, 6-day, and primary myoblasts. Distribution of the motifs across 500 bp from the peak center is reported below. (C) Six4 and Runx1 bind at a subset of 6-day Pax3 loci. Fewer loci were also bound by Jun, Tead1-4, and Usf1 proteins. *k*-means clustering was generated using published ChIP-seq data (from myogenic cell line C2C12). (D) Distribution of ChIP-seq reads for Six4, Jun, Tead1/4, Runx1, and Usf1 1-day and 6-day Pax3 genomic binding data in cluster 1 (from panel C) generated using *k*-means clustering. Curves show overlapping of Six4 and Runx1 binding at Pax3 peaks (±3 kb from peak center). (E) IGV track displaying genomic occupancy for Pax3, Six4, Runx1, Jun, Tead1/4, and Usf1 at the *Cdon* locus. Dashed red square indicates Pax3-bound site characterized by Six4, Runx1, Jun, and Tead1/4 occupancy. (F-G) Knockdown of Six4 or Tead2 in iPax3 PDGFRα+FLK1− cells impairs Pax3 transcriptional activity and ultimately myogenic differentiation. Upon Pax3 induction, day 5 EB cells were analyzed by qRT-PCR with the indicated probes (F) or assayed for terminal differentiation (G). Graph represents mean + SD from at least 3 independent experiments. MYHC (red); nuclei (blue). Bar: 100 μm. *p < 0.05, **p < 0.01, ***p < 0.001. Numerical values are available in S1 Data. AP1, activator protein 1; Cdon, cell adhesion molecule–related/down-regulated by oncogenes; ChIP-seq, chromatin immunoprecipitation sequencing; Ctrl, control; EB, embryoid body; Gapdh, glyceraldehyde 3-phosophate dehydrogenase; IgG, immunoglobulin G; IGV, Integrative Genomics Viewer; iPax3, inducible-Pax3; Lfng, lunatic fringe; Megf10, multiple epidermal growth factor–like domains protein 10; Myf5, myogenic factor 5; MYHC, myosin heavy chain; Pax3, paired box 3; qRT-PCR, quantitative reverse transcription PCR; RFX, regulatory factor X; Runx1, Runt-related transcription factor 1; shSCR, scramble control; shSix4, Six4 knockdown; shTead2, Tead2 knockdown; Six4, sine oculis-related homeobox 4; Tead1/2/4, TEA domain family member 1/2/4; Tot, total; Usf1, upstream stimulatory factor 1.

The studies above strongly support the notion that a major role of Pax3 binding is to promote chromatin remodeling, thereby increasing accessibility of target loci. To identify potential cooperation between Pax3 and other TFs, we analyzed the conserved motifs enriched near Pax3 peaks. To gain a comprehensive assessment of the conserved motifs at Pax3 sites, we performed this analysis on 1-day and 6-day Pax3 ChIP-seq peaks as well as on Pax3 binding sites identified in adult primary myoblasts [51]. This comparison revealed differences in Pax3 genomic binding among these samples (Fig 6A). We found 1,699 sites in common between Pax3 peaks from 1-day and 6-day samples, whereas 504 and 1,184 Pax3 peaks were shared between myoblasts and 1-day and 6-day samples, respectively (Fig 6A), thus suggesting context-dependent binding of Pax3 across different developmental stages. As expected, motif analysis identified the presence of Pax3 binding motifs among the top hits, supporting that our ChIP-seq robustly identified bona fide Pax3 binding sites (Fig 6B). Similar to the NIH3T3-specific Pax3 peaks (Fig 5), Pax3 peaks in myoblasts were found enriched for both paired-type and homeodomain-type motifs (Fig 6B), in agreement with Soleimani and colleagues [51]. In contrast, we detected only paired-type Pax3 binding motifs in 1-day and 6-day peaks (Fig 6B). In addition, we detected several potential binding sites for other TFs, including members of the homeodomain, TEAD, and Runt families, among others (Fig 6B and S7A Fig).

To verify whether the predicted motifs near Pax3 binding sites are occupied by TFs in vivo, we used available ChIP-seq datasets for Six4 [52], Tead1, Tead4 [53], Jun [54], Runt-related transcription factor 1 (Runx1) [55], and upstream stimulatory factor 1 (Usf1) [56]. Six4 is a known regulator of the myogenic lineage, as it binds to the *Myf5* −111 kb and −57 kb enhancers [18,21], and it is required for the MyoD-mediated myogenic conversion of MEFs and muscle differentiation [57,58]. Jun is a component of the activator protein 1 (AP1) factor, which has been shown to bind enhancers in multiple cell types, including skeletal muscle [54,59]. Similarly, Tead and Runx1 factors regulate several myogenic genes in proliferating myoblasts [53,55]. Usf1 has been detected in developing somites [60]. Upon *k*-means clustering of these datasets across a region of ±3 kb from the center of the Pax3 6-day ChIP-seq dataset (Fig 6C and S7 Table), we observed a subset of Pax3 binding sites overlapping with Six4, Tead1/4, Runx1, and Usf1 (Fig 6D: cluster 1). Visual inspection of the genome tracks confirmed the co-occurrence of binding for these TFs (including Jun at selected sites) and Pax3 at subsets of genes such as *Cdon*, *Megf10*, and *Fgfr4* (Fig 6E). We noted that the 4 distinct clusters also differed for recruitment of Pax3 between 1-day and 6-day (Fig 6C: compare clusters 1, 2, and 4 versus cluster 3), indicating that Pax3 binding at a subset of sites changes during the myogenic specification process. In support of the functional relevance of cluster 1 peaks, GO classification using GREAT showed an enrichment for skeletal muscle biological process categories (S7C Fig). Among the other clusters, clusters 2 and 4 displayed an enrichment for myogenic biological process categories, whereas cluster 3 was enriched for terms associated with different aspects of mesoderm specification (S7C Fig). To assess the biological relevance of these TFs during Pax3-induced myogenesis, we performed knockdown studies using constructs targeting Six4 and Tead2, which represents the highest-expressed Tead factor based on our RNA-seq data. Knockdown of Six4 and Tead2 using two independent shRNA clones induced a >50% reduction of the respective mRNA compared to scramble control (shSCR) counterparts (Fig 6F) and resulted in the significant reduction of the expression levels of genes found up-regulated in Pax3-induced EB cultures, including genes associated with Notch and Sonic hedgehog (Shh) signaling pathways (Fig 6F and S7D Fig). We then further investigated whether terminal differentiation of Pax3-induced mesoderm into myosin heavy chain (MYHC+) cells required Six4 or Tead2. Knockdown of both genes significantly reduced differentiation (Fig 6G and S7E Fig), thus confirming that these two TFs participate in the Pax3-mediated activation of the skeletal myogenic program.

Based on these data, we conclude that activation of the skeletal myogenic program during development involves both Pax3-mediated chromatin remodeling and the subsequent recruitment of Six4 and Tead2.

## Discussion

**Fig 7:**
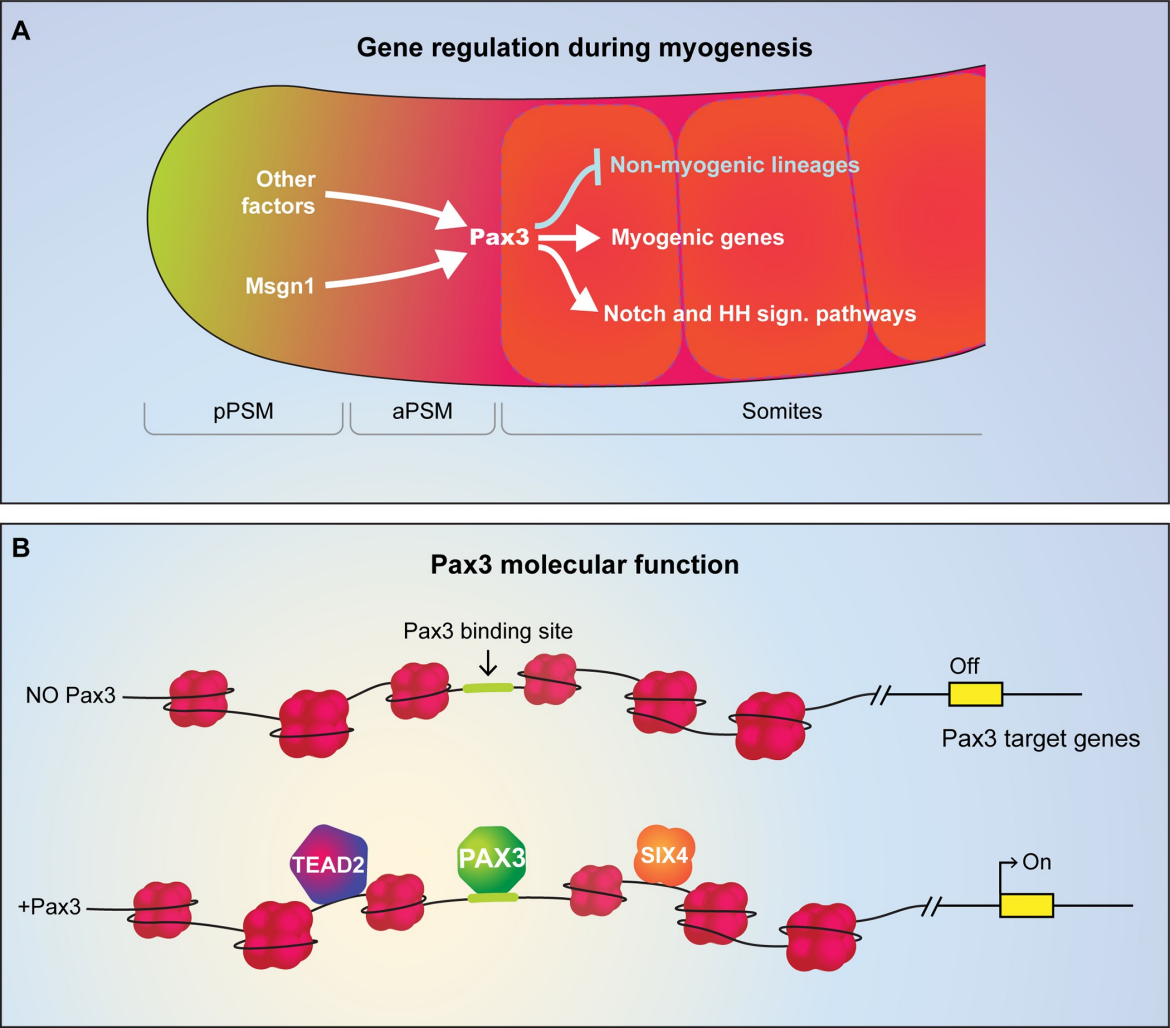
Model of Pax3 function during embryonic development. (A) Pax3 expression is regulated by Msgn1, expressed in the presomitic mesoderm, as well as by other unidentified factors that contribute to its expression in the formed somites. Pax3, in turn, regulates the myogenic program by inducing, among the others, components of Notch and HH signaling pathways while repressing nonmyogenic lineages. (B) Pax3 binds and remodels chromatin by increasing chromatin accessibility. Cooperation between Pax3 and Six4-Tead2 ensures proper activation of the skeletal myogenic program. aPSM, anterior presomitic mesoderm; HH, hedgehog; Msgn1, mesogenin 1; Pax3, paired box 3; pPSM, posterior presomitic mesoderm.

Using genomic techniques combined with in vitro and in vivo models, here we provide the first comprehensive analysis of Pax3-mediated myogenic specification in developing mesoderm. In this study, we identified a compendium of Pax3-bound mesodermal enhancers, defined the epigenetic changes resulting from the recruitment of chromatin remodeling complexes at these sites both in vitro and in vivo, and identified cooperation between Pax3 and the TFs Six4 and Tead2 during myogenic lineage commitment. These analyses provide important insights into what can be defined as the “core function” of Pax3 at its binding sites and its cooperation with other transcriptional regulators (Fig 7).

### TF hierarchy during mesoderm patterning

The use of differentiating inducible ES cell lines expressing TFs involved in mesoderm patterning under serum or serum-free conditions highlights the central role of Pax3 in the specification of the myogenic lineage (Fig 7A). Our data demonstrate that, even though Msgn1 lies upstream of Pax3 in the mesoderm genetic hierarchy [16], it does not efficiently activate the skeletal myogenic program. Nonetheless, we observed that Msgn1 has mitotic bookmarking capability, which we speculate may be involved in Msgn1 role in lineage fate decision of neuromesodermal progenitors. Similar to Msgn1, expression of mesoderm posterior 1 (Mesp1) in serum-free cultures of differentiating mouse ES cells induces partial activation of the skeletal myogenic lineage [26], and in vivo analysis of *mesp-b* function in zebrafish embryos demonstrated this transcriptional regulator is necessary for dermomyotome development [61]. However, although these authors observed that *mesp-b* expression in transgenic embryos directly regulates *meox1* and *ripply1* expression, this was not the case for *pax3*, thus suggesting an indirect role of *mesp-b* in the regulation of this TF. Based on our data, Msgn1 does not induce Mesp1/2, which are up-regulated only upon Tbx6 expression in serum-free condition (S7F Fig). Since partial muscle specification occurs only upon Msgn1 induction under serum-free differentiation conditions, we conclude that other signals are required for the efficient transition of the presomitic progenitors to the myogenic lineage.

In addition, we observed that signaling pathways activated by serum components inhibit Msgn1 paraxial mesoderm inducing activity. In agreement with this hypothesis, Chan and colleagues reported that both Bmp2 and Bmp4 repress Mesp1-mediated myogenic differentiation by promoting the cardiac cell fate [62]. Pax3 function, in contrast, is independent of the growth factor environment, and its induction results in robust activation of the skeletal myogenic program. Because embryonic myogenic progenitors have been suggested not to be affected by the BMP/transforming growth factor beta (TGFβ) pathway [63], we predict this may occur through a Pax3-dependent transcriptional regulation of key components of this pathway during development. Accordingly, in our RNA-seq dataset, we observed down-regulation of *Bmp2/4* and up-regulation of the *Sulf2* and *Twsg1*, secreted factors involved in BMP signaling repression (S3H Fig) [41,64,65], although further studies will be required to investigate whether this process is directly regulated by Pax3. Corroborating our in vitro results, we show the impaired expression of *Meox1* along the dorsoventral axis in *Pax3*-null E9.5 embryos. Previous findings reported for *Paraxis* and *Lfng* in E10.5 splotch-homozygous embryos, a *Pax3*-null allele [66,67], further support the notion that Pax3 regulates paraxial mesoderm specification [25]. Analysis of the ATAC-seq dataset from paraxial mesoderm cells of E9.5 embryos and differentiating inducible Msgn1, Pax3, and Myf5 ES cells revealed that differentially accessible peaks are subdivided into clusters with distinct GO classifications based on the TF of origin. This analysis confirms that Pax3 regulates chromatin accessibility at loci associated with genes involved in the determination of myogenic progenitors and highlights the difference with Myf5, which activates genes further downstream in the muscle differentiation program.

### Pax3-mediated modulation of signaling pathway components

Our transcriptomic and epigenetic studies indicate that Pax3 regulates the expression of a subset of genes associated with HH and Notch signaling pathways. During development, Shh secreted from notochord and dorsal neural tube plays an important role in Myf5 and Myod induction (reviewed by [68]). Cdon, Boc, and growth arrest specific 1 (Gas1) have been identified as essential ligand-binding components of the HH coreceptor Patched [69–71] and are required for proper patterning of multiple tissues [72]. Since Cdon, Boc, and Gas1 are expressed in the Pax3+ dermomyotome [73–75], our data are in agreement with an active and evolutionarily conserved role of Pax3 in binding and remodeling of the regulatory elements responsible for induction of these HH coreceptors. This hypothesis is further supported by the observation that Cdon, Boc, and Gas1 transcripts are up-regulated in the somites of *Pax3*^*Pax3FKHR/GFP*^ embryos, a transcriptionally dominant Pax3 allele [43]. At the molecular level, both Cdon and Boc are part of a cell–cell adhesion complex (which also includes N- and M-cadherins) required for the differentiation of skeletal myogenic progenitors through activation of p38 mitogen-activated protein kinase (MAPK) and cell division cycle 42 (Cdc42) signaling [76]. In accordance, we observed that treatment of Pax3-induced cultures with the p38 inhibitor SB203580 impairs myogenic specification (S7G Fig).

The Notch signaling pathway plays an important role in preventing precocious differentiation of progenitors in the developing muscle (reviewed by [77]). Nonetheless, to date, regulatory networks responsible for the expression of these genes during myogenic specification have not been deeply investigated or have been limited to muscle cell lines, such as C2C12. Our data identify an important function for Pax3 in the expression of components of the Notch pathway. Similar to the HH signaling pathway, Pax3-induced changes in chromatin accessibility were detected at loci associated with Notch pathway genes (e.g., *Lfng* and *Notch2*), as well as extracellular matrix proteins required for the establishment of the future muscle stem cell niche (e.g., *Megf10* and *M-cadherin* [78]). Most of these observations were corroborated by data collected in developing mouse embryos and through analysis of murine and human iPAX3 differentiating ES cultures. Altogether, our data reveal novel Pax3-dependent transcriptional targets accounting for the activation or repression of signaling pathways important for skeletal myogenic lineage specification.

### Pax3 and chromatin remodeling

We demonstrate that Pax3 induction in mesodermal cells results in increased chromatin accessibility at Pax3 targets and epigenetic mark deposition. Histone methylation accounts for diverse functions during transcriptional regulation, and multiple complexes mediate the deposition/erasure of this modification. H3K4me1 and trimethylated lysine 4 of histone 3 (H3K4me3) are deposited by the lysine (K)-specific methyltransferase 2C/D (MLL3/4) and SET1 complexes, respectively, which are characterized by common and specific subunits [79]. Although interaction between the HMT complex and Pax3 has not been reported, studies carried out by Rudnicki and colleagues demonstrated that the Pax7-mediated recruitment of the MLL2-ASH2L-WDR5-RBBP5 complex at the *Myf5* −111 kb and −57 kb enhancers is important for the deposition of H3K4me3 in proximity of the *Myf5* TSS [46,80]. Similarly, other studies focusing on different Pax family members reported their interaction with the MLL3/4 HMT complex [47,48]. Our data showed that Pax3-mediated increased chromatin accessibility at the *Myf5* −111 kb enhancer is associated with deposition of H3K4me1. These findings demonstrating that chromatin accessibility is diminished at Pax3 loci in paraxial mesoderm cells from *Pax3*-null E9.5 embryos support an active role for Pax3 in the regulation of chromatin remodeling during development.

Based on our work and other published studies, we hypothesize that the general function of Pax proteins in transcriptional regulation is to initiate chromatin remodeling at their binding sites, which in turn may enable binding of other TFs participating in a given lineage-specific program, as outlined in Fig 7B for Pax3 and the myogenic lineage. This model is supported by the following observations: (1) Pax3 cooperates with other TFs to activate the myogenic program, as demonstrated by Six4 and Tead2 knockdown; (2) increased chromatin accessibility is observed at Pax3 sites in mesoderm (both in vitro and in vivo) and in NIH3T3 fibroblasts; (3) deposition of H3K4me1 occurs at Pax3 sites also in nonmyogenic cells, such as NIH3T3, even though these cells cannot differentiate into muscle upon Pax3 induction. Of relevance, the related factors Pax7 and Pax2 regulate chromatin accessibility and H3K4me1 deposition in different cell types [45,48,50,81]. Pax7-dependent chromatin remodeling enables T-box 19 (Tpit) binding in pituitary cells [50] and correlates with future MyoD occupancy at a subset of enhancers in myogenic cells [45]. Alignment of the protein sequences of different Pax members identifies high conservation at the N-terminal region corresponding to the DNA-binding domains (paired and homeodomain), whereas the C-terminal regions show very little homology. Although this observation suggests that recruitment of the HMT complex might involve its interaction with the Pax DNA-binding domains, we detected no increase in H3K4me1 deposition using a Pax3 mutant lacking the C-terminal region, thus raising the possibility that the interaction between Pax TFs and the HMT complex might require the interaction with both the N terminus and C terminus of the protein. Future studies investigating the structure of the C-terminal region will be instrumental for addressing this open question.

### Is Pax3 a pioneer TF?

Our data demonstrate that Pax3 modulates chromatin accessibility and H3K4me1 levels, thus establishing a more favorable landscape for the concerted action of cooperating TFs, such as Six4 and Tead2, in the context of myogenic lineage commitment. These findings are compatible with a pioneering function of Pax3 during skeletal myogenic specification, even though additional data would be required to include Pax3 in this category. A recent study demonstrated that pioneer factor binding differs across cell types and that stable occupancy is likely the result of cooperative interactions with other cell-specific TFs [82]. Accordingly, whereas Pax3 expression in NIH3T3 fibroblasts does not activate the skeletal myogenic program and occupies a different subset of loci compared to EB-derived cells, our experiments in NIH3T3 fibroblasts demonstrate that this TF retains the ability to increase chromatin accessibility at a different subset of loci. Coexpression of Pax3, Runx1, Six1, and other mesodermal TFs in NIH3T3 and mouse embryonic fibroblasts (MEFs) does not enhance myogenic commitment, and in fact, other investigators have reprogrammed fibroblasts into myogenic cells solely by Myod transduction [83,84]. Furthermore, in a previous study on Pax7, a closely related Pax gene, we observed that chromatin remodeling occurs primarily at sites depleted of repressive histone marks (trimethylated lysine 27 of histone 3 [H3K27me3] and trimethylated lysine 9 of histone 3 [H3K9me3]; [45]). Based on these data, we speculate that the chromatin status of the cells may represent the main barrier for the Pax3-mediated conversion of nonmesodermal cells into skeletal myogenic progenitors. This hypothesis is in agreement with the enrichment of the CTCF motif at the sites characterized by increased chromatin accessibility upon Pax3 expression in differentiating ES cells, and it is further supported by the observation that pioneer-resistant Pax7-bound loci in melanotrope cells harbor the CTCF sequence [81].

In conclusion, our data provide the first comprehensive analysis of Pax3 function during skeletal myogenesis and demonstrate that Pax3-mediated lineage commitment results from the coordinated modulation of signaling pathways and downstream transcriptional effectors.

## Materials and methods

### Ethics statement

All animals were handled in strict accordance with good animal practice as defined by the relevant national and/or local animal welfare bodies, and all animal work was approved by the University of Minnesota Institutional Animal Care and Use Committee (protocol number 1702-34580A).

### Plasmids

The p2lox-Pax3 vector was previously described [25]. The p2lox-Msgn1 plasmid was generated by subcloning the Msgn1 CDS amplified from genomic DNA (PCR primers available in S8 Table). The Msgn1 PCR product was cloned into the pCR2.1-TOPO vector (Invitrogen) and sequenced prior to subcloning in the final vector. p2lox-Foxc1, p2lox-Meox1, p2lox-Myf5, p2lox-Paraxis, p2lox-Six1, and p2lox-Tbx6 were generated by subcloning the respective coding sequences obtained from Open Biosystems (GE healthcare). The p2lox-Pax3-GFP and p2lox-Msgn1-GFP vectors were generated by subcloning the EGFP sequence at the C-terminal region of Pax3 and Msgn1 cDNA (after removal of the stop codon and separated by a linker, sequence: EFLINAT). Knockdown constructs from the TRC library (pLKO) were obtained from the University of Minnesota Genomic Center (UMGC). pLKO.1-blast-SCRAMBLE was a gift from Keith Mostov (Addgene plasmid #26701 [85]). To purify cells transduced with the knockdown constructs, all pLKO plasmids were modified by replacing the selection gene (puromycin or blasticidin) with the sequence encoding the EGFP (referred hereafter as pLKO-shRNA-PGK-GFP). pSAM2-Pax3-ires-GFP was obtained by subcloning the murine or human Pax3 sequence into the EcoRI site of the pSAM2-ires-GFP lentiviral vector. pFUGW-rtTA and pSAM2-ires-GFP were previously described [86]. Additional cloning details for all constructs will be available upon request.

### Cell cultures

Inducible mouse ES cell lines were generated by Cre-loxP mediated recombination of the p2lox targeting plasmids into A2lox-cre mouse ES cells [23]. The recombination cassette, located next to the Hprt gene, contains the Tet-responsive element (TRE) driving the expression of one single copy of cDNA, thus ensuring quasi-physiological expression levels. Pax3-ires-GFP and control Ires-GFP mES cell lines were described previously [25]. Inducible PAX3 H9 human ES cells were generated by lentiviral transduction of the pSAM2-PAX3-ires-GFP and pFUGW-rtTA constructs and maintained on Matrigel-coated flasks using mTeSR (Stem Cell Technology). mES cells were maintained on mitotically impaired MEFs in KnockOut DMEM (Invitrogen) supplemented with 15% FBS (Embryomax ES-qualified FBS [Millipore]), 1% Penicillin/Streptomycin (Invitrogen), 2 mM GlutaMAX (Invitrogen), 0.1 mM Non-Essential Amino Acids (Invitrogen), 0.1 mM β-mercaptoethanol (Invitrogen), and 1,000 U/ml LIF (Millipore). The myogenic differentiation protocol was previously described [87]. Briefly, the ES/MEF cell suspension was preplated in a gelatin-coated dish for 30 minutes in order to remove fibroblasts, and the resulting supernatant (enriched for mES cells) was then diluted to 40,000 cells/ml in EB differentiation medium and incubated in an orbital shaker at 80 RPM. EB differentiation medium was composed of IMDM (Invitrogen) supplemented with 15% FBS (Embryomax ES-qualified FBS), 1% penicillin/streptomycin (Invitrogen), 2 mM GlutaMAX (Invitrogen), 50 μg/ml ascorbic acid (Sigma-Aldrich), and 4.5 mM monothioglycerol (MP biomedicals). For serum-free differentiation, FBS was replaced with an equivalent amount of KnockOut Serum Replacement (Invitrogen). Transgene induction was achieved by adding dox (Sigma-Aldrich) to day 3 EBs cultures (final concentration 1 μg/ml) and then maintained throughout the differentiation protocol by replacing the media (including dox) every 2 days. At day 5, EBs were disaggregated, and single cells were incubated for 20 minutes with PDGFRα-PE, FLK1-APC, and VCAM1-biotin-conjugated antibodies (e-Bioscience) followed by 5 minutes of incubation with Streptavidin-PeCy7. PDGFRα+FLK1− cells were sorted using FACSAria II (BD biosciences) and replated on gelatin-coated dishes using serum or serum-free EB differentiation media supplemented with 1 μg/ml dox and 10 ng/ml mouse basic FGF (bFGF; Preprotech). Except Myf5+dox cells from serum-free cultures, which did not express PDGFRα and were replated as bulk, analysis of myogenic potential for the other TFs was performed in PDGFRα+FLK1− cells. Skeletal myogenic differentiation was assessed in cells cultured 4 days as monolayer by withdrawing bFGF and dox from the cultures (in order to shut down transgene expression) followed by additional 2 days of culture in the same serum or serum-free media. HEK293T, NIH3T3, and Bend3 cells were maintained in DMEM (Invitrogen) supplemented with 10% FBS (Millipore), 1% penicillin/streptomycin (Invitrogen), and 2 mM GlutaMAX (Invitrogen).

Skeletal myogenic differentiation of H9 iPAX3 ES cells was achieved by plating 10^6^ cells in 6 cm nonadherent Petri dishes and incubated at 37°C, 5% CO_2_ on a shaker at 60 RPM. After 2 days, mTeSR was replaced with EB Myogenic (EBM) Media supplemented with 10 μM Y-27632 and 10 μM GSK3 inhibitor (CHIR990217) (media composition described in [88]). Two days later, media were replaced to remove GSK3 inhibitor, and the formed EBs were plated on gelatin-coated flasks using EBM + 10 ng/ml human bFGF. Twenty-four hours later, cultures were supplemented with 1 μg/ml dox for PAX3 induction. Twenty-four hours later, cells were harvested using trypsin+EDTA solution and FACS sorted based on GFP expression. Sorted cells were collected for RNA extraction. To assess the myogenic commitment upon PAX3 induction, GFP+ cells from H9 iPAX3 differentiating cultures were sorted 4 days post dox supplementation and plated at 1.5 × 10^6^ cells per T25 on gelatin-coated flasks using EBM supplemented with 5 ng/ml bFGF, 1 μg/ml dox, and 5 μM ROCK inhibitor (ROCK inhibitor was removed the day after) and cultured as monolayer. At about 90% cell density, cells were passaged using trypsin+EDTA and replated on new gelatin-coated flasks. Terminal differentiation was induced by switching 100% confluent cultures to KOSR differentiation media (KnockOut DMEM + 20% KnockOut Serum Replacement + 1% penicillin/streptomycin + 2 mM GlutaMAX; all products from Gibco).

### Lentiviral transduction and knockdown

Lentiviruses were produced in HEK293T cells by cotransfection of pVSV-G, Δ8.9 and lentiviral constructs using Lipofectamine LTX-Plus reagent (Invitrogen). Supernatants containing lentiviral particles were filtered using 0.45 μm filters and applied to cells cultured in 6-well plates. To facilitate transduction, 6-well plates were centrifuged for 90 minutes at 1,100*g* at 30°C. Pax3-inducible NIH3T3 and Bend3 cells were generated by cotransduction of pSAM2-Pax3-ires-GFP and pFUGW-rtTA lentiviruses. Six4 and Tead2 knockdown was performed by transducing mES cells with the respective pLKO-shRNA-PGK-GFP constructs (including the shSCR). At day 5 of differentiation, GFP+ cells were sorted using FACSAria II and replated on gelatin-coated dishes or resuspended in Trizol (Invitrogen) for RNA analysis. Replated day 5 PDGFRα+FLK1− cells from Six4 and Tead2 knockdown experiments were cultured 4 days as monolayer followed by withdrawal of bFGF and dox for 2 days before fixation and immunostaining.

### Mice and embryo explants

Pax3-null and Pax3-null;Pax7-het E9.5 embryos were generated by timed mating of Pax3^cre/+^ mice or Pax3^cre/+^;Pax7^+/−^ mice. Time-mated mice were euthanized using CO_2_ for collecting embryos. For RNA analysis and FACS isolation of PDGFRα+FLK1− cells, embryos were dissected under the stereoscope to remove head and heart, and the remaining tissues were dissociated in 0.25% trypsin for 3 minutes at 37°C. Cells from individual embryos were then stained using PDGFRα-PE and FLK1-APC antibodies, FACS sorted, and processed for ATAC-seq. For all experiments, genotyping was performed using genomic DNA isolated from yolk sacs.

### ChIP and library generation

ChIP was performed following the protocol described by Young and colleagues, with minor modifications [89]. D4 EBs (1-day induced and control Pax3 mouse cells) were trypsin-treated at 37°C for 1 minute with gentle shaking, and reaction was inhibited by adding 10% FBS/PBS. One-day induced Pax3 NIH3T3 cells and 6-day induced Pax3 mouse cells growing in monolayer were harvested by incubation with trypsin for 1–2 minutes. Human differentiating PAX3-inducible cells were collected at day 6 of differentiation (following 24-hour dox-mediated induction) by incubation with trypsin for 1–2 minutes. Single cells were washed once with PBS, resuspended in 10% FBS/PBS, and supplemented with formaldehyde (final concentration 1%) for crosslinking of protein-DNA complexes (10 minutes at RT) followed by quenching with glycine and staining with PDGFRα-PE antibody. PDGFRα+ cells were sorted using FACSAria II, snap-frozen in liquid nitrogen, and stored at −80°C if not processed immediately. Cell pellets were incubated in lysis buffer LB1 supplemented with protease inhibitors (50 mM HEPES KOH [pH 7.5], 140 mM NaCl, 1 mM EDTA, 10% glycerol, 0.5% NP40, 0.25% Triton X-100 + complete-mini [Roche]) for 10 minutes at +4°C followed by incubation in buffer LB2 supplemented with protease inhibitors (10 mM Tris-HCl [pH 8], 200 mM NaCl, 1 mM EDTA, 0.5 mM EGTA + complete-mini [Roche]) for 10 minutes at +4°C. Cell pellet was then resuspended in LB3 supplemented with protease inhibitors (10 mM Tris-HCl [pH 8], 100 mM NaCl, 1 mM EDTA, 0.5 mM EGTA, 0.1% Sodium Deoxycholate, 0.5% N-lauroylsarcosine + complete-mini [Roche]) and then sonicated. For H3K4me1, cells were sonicated using Bioruptor Pico (25 cycles 30 seconds ON, 30 seconds OFF) to achieve an average size of 200 bp. For Pax3, cells were sonicated with a Branson sonicator at 18% power for 1 minute with intervals of 10 seconds ON and 10 seconds OFF to achieve an average chromatin size of 300 bp. After shearing, samples were centrifuged for 10 minutes at 16,000*g* and snap-frozen in liquid nitrogen if not processed immediately. For histone ChIP, 12 μg of chromatin (diluted to 250 μl) was precleared for 4 hours at 4°C with 10 μl of BSA-blocked Protein A conjugated sepharose beads (GE Healthcare). For Pax3 ChIP, 25–40 μg of chromatin (diluted to 500 μl) was precleared for 4 hours at 4°C with 20 μl of BSA-blocked Protein A (or Protein G) conjugated sepharose beads (GE Healthcare). Samples were supplemented with 1/10 volume of 10% Triton X-100 and incubated overnight with anti-Pax3 (Santa Cruz Biotechnology sc-34926) or anti-H3K4me1 (ab8895 [Abcam]) antibodies. Immune complexes were recovered by incubation with 20 μl of BSA-blocked Protein G conjugated sepharose beads for 4 hours at 4°C and then washed 5 times with RIPA wash buffer (50 mM HEPES KOH [pH 7.5], 500 mM LiCl, 1 mM EDTA, 1% NP40, 0.25% Triton X-100, 0.7% sodium deoxycholate) and 1 time with TEN buffer (10 mM Tris-HCl [pH 8], 1 mM EDTA, 50 mM NaCl). Immunoprecipitated chromatin was recovered by incubating beads with 200 μl of elution buffer (50 mM Tris-HCl [pH 8], 10 mM EDTA, 1% sodium dodecyl sulfate) for 20 minutes at 65°C. Chromatin from IP and Input (equivalent to 1% of starting material) was reverse cross-linked overnight at 65°C, then diluted 1:1 with TE (10 mM Tris-HCl [pH 8], 1 mM EDTA) supplemented with 4 μl of RNaseA 20 mg/ml, and incubated for 2 hours at 37°C followed by proteinase K treatment (4 μl of 20 mg/ml stock for each sample) for 30 minutes at 55°C. DNA was purified by Phenol-chloroform-isoamyl alcohol extraction (twice) followed by chloroform extraction, then supplemented with 1/10 of volume of 3 M Sodium Acetate (pH 5) and 1.5 μl of glycogen, and precipitated with 2 volumes of 100% ethanol at −80°C for >1 hour. Following 30 minutes of centrifuge at 16,000*g*, pellets were washed with 75% ethanol, air dried, and dissolved in 45 μl H2O. qPCR was performed in a final volume of 10 μl using SYBR Premix Ex Taq II (Clontech), 0.5 μl of 1.4 μM primer stock, and 0.3 μl sample/reaction and run on a 384-well plate on a ABI7900HT instrument (Applied Biosystems). The primer sequences used for qPCR are provided in S8 Table.

Libraries were generated following a gel-free protocol using AMPure XP beads (Beckman Coulter) for all the purification and size selection steps. DNA (10 ng or less) was end repaired using End-It DNA End Repair (Epicentre) and then A-tailed using Klenow Fragment (3′→5′ exo- NEB) followed by adapter-barcode ligation using T4 DNA ligase (Enzymatics). Illumina-compatible adapter barcodes were purchased from BIOO scientific. After ligation, DNAs were negatively size selected using 0.5x AMPure XP beads, and unbound DNAs were positively size selected by adding 0.4x AMPure XP beads (this step allows for retention of DNA fragments ranging 200–500 bp). Libraries were amplified using Phusion High Fidelity PCR master mix 2x (NEB) with a 16-cycle program. Libraries from iPax3 NIH3T3 cells were generated using the NEBNext DNA library prep kit (NEB). Purified libraries were then submitted to the UMGC for quantification, quality control, and sequencing. Libraries were pooled and sequenced on single-end runs of the HiSeq2500 operated at high-output mode (Illumina). Libraries for Pax3 were sequenced to an average depth of 25 million reads per sample. The sequencer outputs were processed using the computer cluster managed by the Minnesota Supercomputing Institute (MSI).

### ChIP-seq analysis

Each sample’s reads were aligned to the mouse (mm10) or human (hg38) genome using Bowtie2 [90], followed by removal of PCR duplicates using Samtools [91]. Peak calling was performed using MACS [92] with the following settings:—bw 300 -p 1e-3. Similar results were obtained by performing peak calling using QESEQ [93] with the following settings: -s 100 -c 15 -p 0.01. To identify the list of high-confidence mouse Pax3 peaks, we performed 3 independent Pax3 ChIP-seq experiments, and, using the BEDTools intersect function, only common regions between 2 experiments were further considered [94]. In addition, the Pax3 peak list was filtered (intersect–v option) for sites overlapping to peaks detected in the uninduced control ChIP-seq and in the mouse ChIP-seq blacklist [56]. Density maps were generated with SeqMiner using the enrichment file enr.sgr produced by QESEQ. Bigwig files for visualization on IGV [95] were generated by converting the wig files obtained from MACS, using the Kent tool Wig-to-BigWig [96]. Motif analysis was performed using the MEME suite [97], but similar results were obtained using the Cistrome tool Seqpos [98].

### ATAC-seq

Analysis of chromatin accessibility was performed following the protocol described by Buenrostro and colleagues [99]. Fifty thousand freshly sorted cells from differentiated ES cultures, iPax3 NIH3T3 fibroblasts, and single embryos were washed with 200 μl of cold PBS and then resuspended in 100 μl of cold lysis buffer (10 mM Tris-HCl [pH 7.4], 10 mM NaCl, 3 mM MgCl2, 0.1% IGEPAL CA-630), spun at 500*g* for 10 minutes at 4°C, and resuspended in 50 μl of the transposition reaction mix. Transposition occurred at 37°C for 30 minutes, after which transposed DNA was purified using a Qiagen MinElute Kit and eluted in 10 μl elution buffer. PCR amplification using Illumina-compatible adapter barcodes and final library preparation was performed at the UMGC. After quality control, libraries were pooled and sequenced on paired-end runs of the HiSeq2500 operated at high-output mode (Illumina).

### ATAC-seq analysis

Reads were aligned to the mouse genome (mm10) using Bowtie2 with the following settings: -q -I 38 -X 2000—local—dovetail—no-mixed—no-discordant. Aligned reads were filtered for PCR duplicates using Samtools and then processes with MACS 1.4 with the following settings:—nomodel—nolambda—keep-dup all—call-subpeaks -p 1e-4 -w–S. Bigwig files for visualization on IGV were generated by converting the wig files obtained from MACS, using the Kent tool Wig-to-BigWig. ATAC-seq reads overlapping to Pax3 peaks were retrieved using the R Bioconductor package and then used to generate density maps with Seqminer [100]. To identify differentially accessible peaks, we used the BEDTools intersect function: the common regions (-f 0.5 -F 0.5 -e options) for the dox-induced samples were intersected (-v -f 0.2 options) with the combined list of control peaks (from noninduced cells), and the resulting list was then compared (-f 0.5 -F 0.5 –e options) with the peak list detected in PDGFRα+FLK1− cells from E9.5 embryos. Motif analysis was performed using the MEME suite, but similar results were obtained using the Cistrome tool Seqpos. To generate ATAC-seq heatmaps, peaks associated with specific samples were combined into a single BED file for each set of sample of interest: (1) Msgn1-only, Pax3-only, and Myf5-only peaks in serum-free condition and (2) 1-day-only and 6-day-only in serum condition. The average coverage across all regions in the combined BED files was calculated by BEDTools map for each sample from coverage information in the BEDGRAPH files generated by MACS2. Heatmaps and clusters were created using R v3.5.1 and the pheatmap package from combined coverage data via a custom R script. Heatmaps show scaled (*z* score) coverage information for each region in the relevant BED file. Clusters were created using euclidean distance measurements and the R function cutree to get a specified number of clusters equal to the number of sample types. Functional annotation of differentially accessible peaks was performed using GREAT (http://bejerano.stanford.edu/great/public/html/) [33]. The GREAT association rule was the following: Single nearest gene: 500 kb max extension, curated regulatory domains included.

### Western blot and immunoprecipitation

Proteins were extracted from cultured cells using RIPA buffer (150 mM NaCl, 50 mM Tris-HCl [pH 7.5], 1 mM EDTA, 1% Triton, 1% Sodium Deoxicholate, 0.1% SDS) supplemented with protease inhibitors (Complete [Roche]) and quantified with Bradford reagent (Sigma-Aldrich). Protein samples were prepared in Laemmli buffer and loaded on gels for SDS-PAGE. Proteins were transferred on PVDF membranes (Millipore) for the detection with the indicated antibodies. A list of antibodies used in this study is provided in S8 Table.

### RNA isolation, qRT-PCR, and transcriptomic analysis

Samples were resuspended in Trizol (Invitrogen) prior to RNA isolation using the PureLink RNA Mini kit (Invitrogen) following the manufacturer’s instructions for “Trizol samples” (including in-column DNase treatment). RNAs were retrotranscribed using Superscript Vilo (Invitrogen). Gene expression analyses were performed using an amount of cDNA corresponding to 12.5 ng of starting RNA for each reaction. qRT-PCR was performed using Premix Ex Taq Probe qPCR Master Mix (Takara) and TaqMan probes (Applied Biosystems).

The 1-day and 6-day transcriptomic analyses were performed on differentiating iPax3 cells. Noninduced and 1-day induced samples represent PDGFRα+FLK1− FACS-sorted cells from day 4 EBs. Six-day samples represent PDGFRα+FLK1− FACS-sorted cells from day 5 EBs, which were further cultured as monolayer for additional 4 days. Libraries were generated using the TrueSeq stranded kit and dual-index adapter barcodes at the UMGC. The libraries were then pooled and sequenced on a paired-end run on the NovaSeq (Illumina). FastQ paired-end reads (150 bp, *n* = 12.8 million per sample) were trimmed using Trimmomatic (v0.33) enabled with the “-q” option; 3 bp sliding-window trimming from the 3′ end requiring minimum Q30. Quality control on raw sequence data for each sample was performed with FastQC. Read mapping was performed via Hisat2 (v2.0.2) using the mouse genome (mm10) as a reference. Gene quantification was done via Cuffquant for FPKM values and feature counts for raw read counts. Differentially expressed genes were identified using the edgeR (negative binomial) feature in CLCGWB (Qiagen, Valencia, CA, United States) using raw read counts. We filtered the generated list based on a minimum 2X absolute fold change and FDR-corrected *p* < 0.05. The R heatmap.3 function was used to display the resulting heatmap. Functional annotation of differentially expressed genes was performed using the online tool DAVID [101]. Graphs represent the output of biological process category.

The transcriptomic analysis of human H9 iPAX3 cells was performed on day 6 of differentiation. Noninduced cells were collected by trypsinization and sorted by FACS to eliminate dead cells (based on PI staining). Twenty-four-hour dox-treated H9 iPAX3 cells were FACS purified based on GFP expression (from PI-negative gate). Total RNA (100 ng) from 3 independent replicates/condition was used to generate libraries using the ligation-mediated sequencing (LM-Seq) protocol [102] and quantitated using the Qubit fluorometer (Life Technologies) following the author’s instructions. The libraries were then pooled using 13 ng/sample for a 51+10 cycle single-read run on the HiSeq 2500 (Illumina) by high-output sequencing. The sequencer outputs were processed using Illumina’s CASAVA-1.8.2 base-calling software. Each sample’s reads were then processed using RSEM version 1.2.3 (with bowtie-0.12.9 for the alignment step) [103,104]. Genes were filtered to select only those with median-normalized transcripts per million mapped reads (TPM) greater than 10 in at least one sample, log2fold change greater than 1, and *p* < 0.05. The R heatmap.3 function was used to display the resulting heatmap. Functional annotation of differentially expressed genes was performed using the online tool DAVID [101]. Graphs represent the output of biological process category.

### Immunostaining

Immunofluorescence staining was performed by fixing cells with 4% paraformaldehyde (PFA)/PBS for 10 minutes at 4°C, then permeabilizing them with 0.1% Triton/PBS, and blocking with 5% BSA/PBS before incubating with the primary antibodies. Samples were rinsed with PBS, blocked with 5% BSA/PBS, and then incubated with the secondary antibody. After washing, samples were mounted on the slides using Prolong Gold with DAPI (Invitrogen). Pictures were acquired with Axioimager M1 fluorescence microscope and quantified using the ImageJ distribution Fiji [105]. Analysis of myogenic differentiation potential of inducible-TF mES cells was performed as follows: color channels were separated, and threshold levels for the red and blue channels were adjusted in order to select the area positive respectively to MYOG or MYHC (red) and DAPI (blue). The area positive for each channel was analyzed using Analyze Particle using 0–Infinity as the size setting. Finally, the percentage of MYOG+ or MYHC+ area for each image was normalized based on nuclear staining (DAPI+). Data represent mean ± SD of representative pictures from 3 independent experiments.

### Fluorescence live cell imaging

PDGFRα+FLK1− sorted cells (serum or serum-free) expressing dox-inducible Msgn1-GFP or Pax3-GFP fusion proteins were plated on Matrigel-coated 35 mm glass-bottom dishes (Ibidi). Twenty-four hours after plating, living cells were incubated with 5 μg/ml Hoechst 33342 for 15 minutes and subsequently analyzed using a Nikon inverted TiE deconvolution microscope system. Cells were kept in a live cell environmental chamber (Ibidi) during image acquisition (37°C, 5% CO_2_, and 90% humidity). Phenol red–free basal medium was used to decrease background fluorescence. At least 10 images were collected and analyzed from 3 independent cell preparations for each TF-GFP expressing line. Following acquisition, images were deconvolved using the NIS-Elements deconvolution module. Cells expressing H2B-GFP or Pax3 (no GFP) were used as positive and negative controls, respectively, for imaging analysis purposes.

### Whole-mount in situ hybridization

mRNA was stained by in situ hybridization [106]. Embryos were dissected in cold 1xPBS, fixed overnight in 4% PFA at 4°C, and dehydrated in methanol. Following rehydration to 1xPBS 0.1%Tween-20 (PTw), embryos were incubated in 6% hydrogen peroxide, 10 minutes in 10 μg/ml proteinase K, postfixed for 30 minutes in 4% PFA, 0.1% glutaraldehyde, and transferred to hybridization solution (50% formamide, 5xSSC, 0.5% CHAPS, 100 μg/ml Heparin, 0.2% Tween-20, 50 μg/ml yeast tRNA). After 1 hour at 65°C, digoxigenin (DIG)-labeled riboprobe was added to 1 μg/ml and embryos incubated for 18 hours. Meox1 probe sequence was previously described [107]. Following hybridization, embryos were washed twice in 5xSSC, 1% SDS for 10 minutes; transferred to 200 mM NaCl, 50 mM Tris (pH 7.5) with 1 U/ml RNaseA for 30 minutes; and washed twice for 30 minutes in 2xSSC. Embryos were blocked in 2% ‎Roche Blocking reagent (RBr; 11096176001 ROCHE) in TBST for 3 hours and incubated in 0.2% RBr at 4°C overnight 1:2,000 dilution of AP-anti-DIG (11093274910 ROCHE). Embryos were then washed 10 times in TBST for 24–48 hours. Embryos were next transferred to NTMT (100 mM NaCl, 100 mM Tris [pH 9.5], 50 mM MgCl2, 0.1% Tween-20) and color developed by incubating in the dark in with NBT/BCIP (Promega). After extensive washing in PTw with 1 mM EDTA, embryos were clarified in 80% glycerol and photographed on a dissecting microscope.

### Statistical analysis

Differences between multiple samples in Fig 1B and 1C, S5C Fig, and S7E Fig were analyzed for statistical significance using one-way ANOVA. Statistical analyses between control and treated groups in Figs 3G, 5B and 6F and S3B, S3D, S3H, S6A, S7D and S7F Figs were performed using paired two-tailed Student *t* test. Statistical analysis reported in Fig 1F and S5G Fig were assessed using unpaired two-tailed Student *t* test. *p*-Values < 0.05 were considered to be statistically significant.

### Genomic datasets

Sequencing data generated in this work are available from the GEO database under the accession number GSE125203. The following genomic data were also used in this study: microarray embryos (GSE22040); Jun (GSE37525), Runx1 (GSE56077); Six4 (GSE6690); Tead1 and 4 (GSE82193); Msgn1 (GSE55263), Usf1 (GSE49847), Pax3 –myoblasts (GSE25092).

## Supporting information

S1 Fig (Related to Fig 1). Distinct functions of Msgn1, Pax3, and Myf5 during mesoderm specification. (A) qPCR validation of TFs induction. Fold induction (+dox/no dox) is reported below. (B) FACS plots of day 5 EBs from A2lox-Pax3, A2lox-FoxC1, A2lox-Meox1, A2lox-Msgn1, A2lox-Myf5, A2lox-Paraxis, and A2lox-Six1 ES cell lines differentiated in serum and serum-free conditions. y-axis: FLK1; x-axis: PDGFRα. (C) qPCR validation of selected genes in day 5 EBs from serum- and serum-free differentiation. Graph represents mean + SD from 2 or 3 independent experiments. (D) Western blot of day 10 cultures from serum- and serum-free differentiation of A2lox-Pax3, A2lox-FoxC1, A2lox-Meox1, A2lox-Paraxis, and A2lox-Six1 ES cell lines. eMYHC. MYOG. ACTIN. (E) Expression of Eya1-4 and Myf5 genes in noninduced, 1-day, and 6-day Pax3 induced cells. Graph represents mean+SD of expected counts in logarithmic scale. Data were pulled from RNA-seq reported in Fig 3A and 3B. (F) FACS plots of day 5 EBs from A2lox-Pax3-GFP and A2lox-Msgn1-GFP ES cell lines differentiated in serum-free condition. y-axis: FLK1; x-axis: PDGFRα. (G) Immunofluorescence staining for MyoG in FACS-sorted PDGFRα+FLK1− cells from serum-free day 10 cultures following 24 hours of dox withdrawal. Images are representative of 3 biological replicates. MYOG (red); nuclei (blue). Bar: 100 μm. (H) Live cell imaging of Pax3, H2B-GFP, Msgn1-GFP, and Pax3-GFP fusion proteins using wide-field microscopy followed by image deconvolution. DNA was visualized using Hoechst 33342. Bar: 5 μm. Numerical values are available in S1 Data. dox, doxycycline; EB, embryoid body; eMYHC, embryonic myosin heavy chain; ES, embryonic stem; FACS, fluorescence-activated cell sorting; FoxC1, forkhead box C1; Meox1, mesenchyme homeobox 1; Msgn1, mesogenin 1; Myf5, myogenic factor 5; MYOG, myogenin; Pax3, paired box 3; PDGFRα, platelet-derived growth factor alpha; qPCR, quantitative PCR; RNA-seq, RNA sequencing; Six1, sine oculis-related homeobox 1; TF, transcription factor.
(TIF)

S2 Fig (Related to Fig 2). Analysis of ATAC-seq data from iMsgn1, iPax3, and iMyf5 ES cell lines and PDGFRα+FLK1− cells isolated from the trunk region of E9.5 mouse embryos. (A) Representative IGV tracks for genes associated with paraxial mesoderm/somite formation, myogenic progenitor specification, and muscle differentiation and comparison with PDGFRα+FLK1− cells isolated from E9.5 mouse embryos. (B) Heatmap displaying the changes in chromatin accessibility in PDGFRα+FLK1− cells from E9.5 embryos and noninduced, Msgn1-, Pax3-, and Myf5-induced cells from serum-free differentiation. Differential accessible loci from the comparison of each TF versus noninduced cells were combined in a list of unique peaks and used to generate the differential analysis. Five clusters (indicated on the right side) were identified, and the corresponding coordinates were used for GO analysis. Legend indicates the scaled (*z* score) coverage information for each region. (C) IGV track displaying chromatin accessibility at the *Myog* locus in cells isolated from 1-day and 6-day Pax3-induced (+) and noninduced (-) EB cultures. Dashed red squares show increased chromatin accessibility at the *Myog* promoter. This region is a known binding site for muscle regulatory factors. DNase-seq data for E9.5 and E10.5 embryos from Encode consortium are shown below. (D–F) Schematic tables reporting outputs from MEME motif analyses for Msgn1-, Pax3-, and Myf5-induced peaks in serum-free differentiation. (G) ChIP-qPCR validation of Msgn1 binding to the Pax3 locus. Graph represents mean + SD of at least 3 independent biological replicates. *p < 0.05, **p < 0.01. (H) Western blot analysis of MSGN1 expression in Msgn1-induced cultures following 1-day and 6-day doxycycline treatment. GAPDH was used as loading control. Numerical values are available in S1 Data. ATAC-seq, assay for transposase-accessible chromatin sequencing; ChIP, chromatin immunoprecipitation; E, embryonic day; EB, embryoid body; ES, embryonic stem; GAPDH, glyceraldehyde 3-phosphate dehydrogenase; GO, gene ontology; IGV, Integrative Genomics Viewer; iPax3, inducible-Pax3; Msgn1, mesogenin 1; Myf5, myogenic factor 5; Pax3, paired box 3; qPCR, quantitative PCR; RNA-seq, RNA sequencing; TF, transcription factor.
(TIF)

S3 Fig (Related to Fig 3). PAX3 transcriptional changes in differentiating human ES cells. (A) Heatmap of genes up-regulated upon 1-day and 6-day Pax3 induction in mouse cells. Changes are relative to noninduced iPax3. A subset of 1-day induced genes is down-regulated in 6-day samples. Selected affected by Pax3 are indicated on the right side of the heatmap. (B) qPCR validation of selected genes from Fig 3. Graph represents mean + SD of at least 3 independent biological replicates. *p < 0.05, **p < 0.01, ***p < 0.001. (C) Immunofluorescence staining for MYOG and MYHC in terminally differentiated cultures from PAX3-induced H9 cells. Left: MYOG (red). Right: MYHC (red). Nuclei (blue). Bar: 100 μm. (D) qPCR analysis of selected genes upon 24 hours of PAX3 expression in differentiating H9 cells. Cells were collected at day 6 of differentiation. Graph represents mean + SD of at least 3 independent biological replicates. *p < 0.05, **p < 0.01. (E) Heatmap of genes up-regulated by PAX3 on day 6 differentiating H9 cells from dox-treated and untreated cultures. (F) Gene ontology analysis of PAX3-up-regulated genes using DAVID. (G) Venn diagram displaying overlap among differentially expressed genes in 1-day and 6-day mouse and 1-day human cells upon Pax3 induction. (H) Gene expression data for Bmp2, Bmp4, Sulf2, and Twsg1 extracted from RNA-seq analysis of Pax3-induced (+dox) and noninduced (no dox) differentiating mouse and human ES cells. Bars represent fold induction (+dox/no dox) of each sample’s mean. *p < 0.05, **p < 0.01, ***p < 0.001. Numerical values are available in S1 Data. Bmp, bone morphogenetic protein; dox, doxycycline; ES, embryonic stem; iPax3, inducible-Pax3; MYHC, myosin heavy chain; MYOG, myogenin; Pax3, paired box 3; qPCR, quantitative PCR; RNA-seq, RNA sequencing; Sulf2, sulfatase 2; Twsg1, twisted gastrulation BMP signaling modulator 1.
(TIF)

S4 Fig (Related to Fig 4). Pax3 genomic occupancy in differentiating mouse and human ES cells. (A) qPCR validation of selected Pax3-bound loci identified by ChIP-seq in day 4 EBs. Mean + SD of at least 3 independent biological replicates is shown. (B) IGV tracks displaying Pax3 genomic occupancy at target gene loci (*Dbx1*, *Ebf3*, and *Prrx1*) in 1-day and 6-day Pax3 ChIP-seq murine datasets. Dashed red squares indicate Pax3 peaks. (C) GREAT functional classification based on biological process of 1-day (3,780) and 6-day (5,710) Pax3 ChIP-seq peaks. Complete annotation data are reported in S2 Table. (D) IGV tracks displaying conserved PAX3 genomic occupancy at loci encoding for Hedgehog (*CDON* and *GAS1*) and Notch (*NOTCH2*) genes in the human genome. Conservation across species and peaks from the 1-day Pax3 ChIP-seq murine dataset (upon genome batch conversion to hg38) are shown below the PAX3 ChIP-seq from differentiating human ES cells. Dashed red squares indicate conserved Pax3 peaks. Numerical values are available in S1 Data. ChIP-seq, chromatin immunoprecipitation sequencing; EB, embryoid body; ES, embryonic stem; IGV, Integrative Genomics Viewer; Pax3, paired box 3; qPCR, quantitative PCR.
(TIF)

S5 Fig (Related to Fig 4). Pax3 regulates chromatin accessibility during myogenic commitment. (A) IGV track displaying chromatin accessibility and Pax3 genomic binding at the *Myod* locus in cells isolated from 1-day and 6-day Pax3-induced (+) and noninduced (-) EB cultures. Dashed red squares show Pax3-dependent regulation of chromatin accessibility at several Pax3-bound sites. Pax3 binding to these elements are shown by the ChIP-seq tracks. (B) GREAT functional annotation of genomic loci in clusters 1, 2, and 3 from chromatin accessibility analysis of noninduced, 1-day, and 6-day Pax3-induced cells (from Fig 4B). Graphs report biological process classification. Complete annotation data are reported in S2 Table. (C) qRT-PCR analysis of selected genes following 1-day and 6-day Pax3-induction. Mean + SD of at least 3 independent biological replicates is shown. *p < 0.05, **p < 0.01, ***p < 0.001. (D) *k*-means clustering of ATAC-seq data from PDGFRα+FLK1− cells isolated from Pax3 1-day induced (+) and noninduced (-) day 4 EBs and Pax3 WT and KO E9.5 embryos. Graph represents the ATAC-seq reads overlapping to 1-day Pax3 ChIP-seq peaks (Pax3 peak center ± 3 kb). (E) Distribution of ATAC-seq reads in clusters 1 and 3 from *k*-means clustering (panel F). (F) IGV track displaying chromatin accessibility at the *Vcam1* and *Myf5* loci in PDGFRα+FLK1− cells isolated from Pax3-induced (+) and noninduced (-) day 4 EBs and Pax3 WT and KO E9.5 embryos. Dashed red square shows Pax3-dependent regulation of chromatin accessibility at the Pax3-bound *Vcam1* +3 kb and *Myf5* −111 kb sites. Pax3 binding to *Vcam1* +3 kb region and *Myf5* −111 kb are shown by the ChIP-seq track. (G) qRT-PCR analysis of selected genes in trunk explants (somite + neural tube) from Pax3-null (KO: *n* = 4) and WT (*n* = 3) E9.5 embryos. Graph represents mean + SD of independent biological replicates. *p < 0.05, **p < 0.01, ***p < 0.001. Numerical values are available in S1 Data. ATAC-seq, assay for transposase-accessible chromatin sequencing; ChIP-seq, chromatin immunoprecipitation sequencing; E, embryonic day; EB, embryoid body; IGV, Integrative Genomics Viewer; KO, knockout; Pax3, paired box 3; qRT-PCR, quantitative reverse transcription PCR; WT, wild-type.
(TIF)

S6 Fig (Related to Fig 5). Pax3 does not activate the myogenic program in NIH3T3 fibroblasts and Bend3 endothelial cells. (A) Analysis of H3K4me1 deposition across the *Myf5* locus in control (no dox) and Pax3-induced (+dox) day 4 EB, NIH3T3, and Bend3 cells. Graph represents mean + SD from at least 3 independent experiments. *p < 0.05. (B) GREAT functional annotation of peaks from EB-only and NIH3T3-only Pax3 ChIP-seq (from Fig 5D). Graphs report biological process classification. Complete annotation data are reported in S2 Table. (C-E) Schematic tables reporting outputs from MEME motif analyses for EBs-only peaks, common EBs/NIH3T3 peaks, and NIH3T3-only peaks. (F) *k*-means clustering of ATAC-seq data from 1-day induced (+) and noninduced (-) EB-derived and NIH3T3 iPax3 cells. Graph represents the ATAC-seq reads overlapping to EBs-only, common EBs/NIH3T3, and NIH3T3-only Pax3 ChIP-seq peaks (Pax3 peak center ± 3 kb). (G) Distribution of ATAC-seq reads in clusters 1 and 3 from panel F. Curves show chromatin accessibility centered on EBs-only, common EBs:NIH3T3, and NIH3T3-only Pax3-bound peaks. Datasets are independent biological replicates. Graph represents the ATAC-seq reads overlapping to Pax3 ChIP-seq peaks (Pax3 peak center ± 3 kb). Numerical values are available in S1 Data. ATAC-seq, assay for transposase-accessible chromatin sequencing; ChIP-seq, chromatin immunoprecipitation sequencing; dox, doxycycline; EB, embryoid body; H3K4me1, monomethylated lysine 4 of histone 3; iPax3, inducible-Pax3; Pax3, paired box 3.
(TIF)

S7 Fig (Related to Fig 6). Pax3 cooperates with Six4 and Tead2 to activate the myogenic program. (A) Selected transcription factor motifs enriched at Pax3-bound loci from 1-day, 6-day, and primary myoblasts. Distribution of the motifs across 500 bp from the peak center is reported below. (B) IGV track displaying genomic occupancy for Pax3, Six4, Runx1, Jun, Tead1/4, and Usf1 at the *Megf10* and *Fgfr4* loci. Dashed red square indicates Pax3-bound site characterized by Six4, Runx1, Jun, and Tead1/4 occupancy. (C) GREAT functional classification of loci from clusters 1, 2, 3, and 4 shown in Fig 6C. Complete annotation data are reported in S2 Table. (D) Gene expression analysis of day 5 EB cells upon Six4 and Tead2 knockdown. Indicated transcripts were analyzed by qRT-PCR with the indicated probes. Graph represents mean + SD from at least 3 independent experiments. (E) Quantification of the MYHC+ area of the immunostaining images shown in Fig 6G. Graph represents mean + SD from at least 3 independent experiments. ***p < 0.001. (F) Expression analysis of Mesp1-2 in nontreated (no dox) and induced (+dox) day 5 EB cells from inducible Msgn1 and Tbx6 ES cell lines differentiated in serum and serum-free conditions. Graph represents mean + SD from at least 3 independent experiments. *p < 0.05, **p < 0.01. (G) Western blot of day 10 cultures from serum differentiation of iPax3 cells. Day 5 EB-derived PDGFRα+FLK1− sorted cells from Pax3-induced (+) and noninduced (-) cultures were treated with the p38 inhibitor SB203580 or the vehicle (DMSO) and collected for analysis after 5 days. eMYHC. MYOG. ACTIN. Numerical values are available in S1 Data. dox, doxycycline; EB, embryoid body; ES, embryonic stem; eMYHC, embryonic MYHC; IGV, Integrative Genomics Viewer; iPax3, inducible-Pax3; Mesp1-2, mesoderm posterior 1–2; Msgn1, mesogenin 1; MYHC, myosin heavy chain; MYOG, myogenin; Pax3, paired box 3; qRT-PCR, quantitative reverse transcription PCR; Runx1, Runt-related transcription factor 1; Tead, TEA domain family member; Tbx6, T-box 6; Usf1, upstream stimulatory factor 1.
(TIF)

S1 Table Genomic coordinates (mm10) of loci identified by ATAC-seq (relative to Figs 2 and 4 and S2B Fig). ATAC-seq, assay for transposase-accessible chromatin sequencing.
(XLSX)

S2 Table GO analysis results from GREAT (related to Fig 2, S2, S4, S5, S6 and S7 Figs). GO, gene ontology.
(XLSX)

S3 Table List of differentially expressed genes following Pax3 induction (1-day and 6-day) in differentiating mouse ES cells and GO analysis from DAVID (related to Fig 3). ES, embryonic stem; GO, gene ontology; Pax3, paired box 3.
(XLSX)

S4 Table Common differentially expressed genes in mouse and human transcriptomic data (related to S3 Fig). (XLSX)

S5 Table Genomic coordinates of Pax3 binding sites in mouse (mm10) and human (hg38) cells (related to Fig 4 and S4 Fig). Pax3, paired box 3.
(XLSX)

S6 Table Annotation of murine Pax3 binding sites to the nearest gene using PAVIS (related to Fig 4). Pax3, paired box 3.
(XLSX)

S7 Table Genomic coordinates (mm10) of Pax3-bound loci in clusters 1–4 (related to Fig 6). Pax3, paired box 3.
(XLSX)

S8 Table List of primers and antibodies used in this study. (XLSX)

S1 Data Numerical values underlying the summary data displayed in this study. (XLSX)

## Abbreviations

ADAM: a disintegrin and metallopeptidase domain 10
AP: anterior–posterior
AP1: activator protein 1
aPSM: anterior presomitic mesoderm
ATAC-seq: assay for transposase-accessible chromatin sequencing
bHLH: basic helix-loop-helix
BMP: bone morphogenetic protein
Boc: biregional Cdon binding protein
Cdc42: cell division cycle 42
Cdon: cell adhesion molecule–related/down-regulated by oncogenes
ChIP-seq: chromatin immunoprecipitation sequencing
*Ckm*: *muscle creatine kinase*
CTCF: CCCTC-binding factor
DLL: delta like canonical Notch ligand
dox: doxycycline
E: embryonic day
EB: embryoid body
eMYHC: embryonic myosin heavy chain
ES: embryonic stem
excr: excretion
Eya: eyes absent
FACS: fluorescence-activated cell sorting
Foxc1: forkhead box C1
Gapdh: glyceraldehyde 3-phosophate dehydrogenase
Gas1: growth arrest specific 1
GFP: green fluorescent protein
GO: gene ontology
H3K4me1: monomethylated lysine 4 of histone 3
H3K4me3: trimethylated lysine 4 of histone 3
H3K9me3: trimethylated lysine 9 of histone 3
H3K27me3: trimethylated lysine 27 of histone 3
HH: hedgehog
HMT: histone methyltransferase
ICN: intracellular notch
IgG: immunoglobulin G
iPax3: inducible-Pax3
KO: knockout
MAML: Mastermind-like
MAPK: mitogen-activated protein kinase
MEF: mouse embryonic fibroblast
MEF2: myocyte enhancer factor 2
Megf10: multiple epidermal growth factor–like domains protein 10
Meox: mesenchyme homeobox
Mesp1: mesoderm posterior 1
MLL3/4: lysine (K)-specific methyltransferase 2C/D
morph: morphology
MRF: muscle regulatory factor
Msgn1: mesogenin 1
Myf5: myogenic factor 5
MyoD: myogenic differentiation protein
MyoG: myogenin
organiz: organization
Pax: paired box
PDGFRα: platelet-derived growth factor receptor alpha
pos reg: positive regulation
pos regul transcr RNA pol II prom: positive regulation of transcription from RNA pol II promoter
pPSM: posterior presomitic mesoderm
RFX: regulatory factor X
RNA-seq: RNA sequencing
Runx1: Runt-related transcription factor 1
Shh: Sonic hedgehog
shSCR: scramble control
Six: sine oculis-related homeobox 1
Six4: sine oculis-related homeobox 4
SMO: Smoothened
SSRP1: structure-specific recognition protein
Tbx6: T-box 6
Tead: TEA domain family member
TF: transcription factor
TGFβ: transforming growth factor beta
Tpit: T-box 19
TSS: transcription start site
Usf1: upstream stimulatory factor 1
Vcam1: vascular cell adhesion molecule 1
WT: wild-type
