# Supplementary figures and images for: Time-dependent Pax3-mediated chromatin remodeling and cooperation with Six4 and Tead2 specify the skeletal myogenic lineage in developing mesoderm
Source: PLoS Biol. 2019 Feb 26;17(2):e3000153. doi: 10.1371/journal.pbio.3000153 (PMC6390996; doi:10.1371/journal.pbio.3000153)

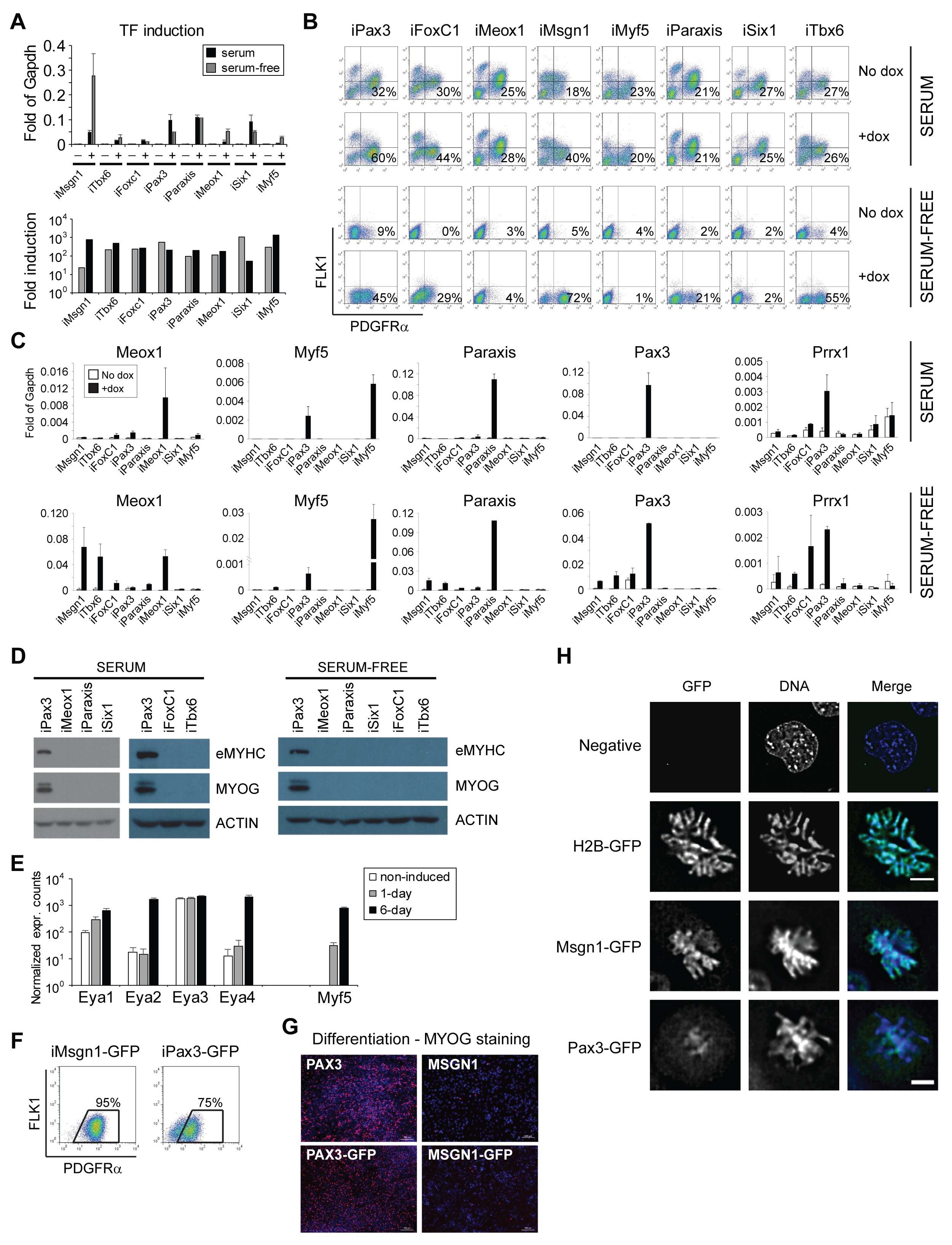

Supplement: S1 Fig — Distinct functions of Msgn1, Pax3, and Myf5 during mesoderm specification. (A) qPCR validation of TFs induction. Fold induction (+dox/no dox) is reported below. (B) FACS plots of day 5 EBs from A2lox-Pax3, A2lox-FoxC1, A2lox-Meox1, A2lox-Msgn1, A2lox-Myf5, A2lox-Paraxis, and A2lox-Six1 ES cell lines differentiated in serum and serum-free conditions. y-axis: FLK1; x-axis: PDGFRα. (C) qPCR validation of selected genes in day 5 EBs from serum- and serum-free differentiation. Graph represents mean + SD from 2 or 3 independent experiments. (D) Western blot of day 10 cultures from serum- and serum-free differentiation of A2lox-Pax3, A2lox-FoxC1, A2lox-Meox1, A2lox-Paraxis, and A2lox-Six1 ES cell lines. eMYHC. MYOG. ACTIN. (E) Expression of Eya1-4 and Myf5 genes in noninduced, 1-day, and 6-day Pax3 induced cells. Graph represents mean+SD of expected counts in logarithmic scale. Data were pulled from RNA-seq reported in Fig 3A and 3B. (F) FACS plots of day 5 EBs from A2lox-Pax3-GFP and A2lox-Msgn1-GFP ES cell lines differentiated in serum-free condition. y-axis: FLK1; x-axis: PDGFRα. (G) Immunofluorescence staining for MyoG in FACS-sorted PDGFRα+FLK1− cells from serum-free day 10 cultures following 24 hours of dox withdrawal. Images are representative of 3 biological replicates. MYOG (red); nuclei (blue). Bar: 100 μm. (H) Live cell imaging of Pax3, H2B-GFP, Msgn1-GFP, and Pax3-GFP fusion proteins using wide-field microscopy followed by image deconvolution. DNA was visualized using Hoechst 33342. Bar: 5 μm. Numerical values are available in S1 Data. dox, doxycycline; EB, embryoid body; eMYHC, embryonic myosin heavy chain; ES, embryonic stem; FACS, fluorescence-activated cell sorting; FoxC1, forkhead box C1; Meox1, mesenchyme homeobox 1; Msgn1, mesogenin 1; Myf5, myogenic factor 5; MYOG, myogenin; Pax3, paired box 3; PDGFRα, platelet-derived growth factor alpha; qPCR, quantitative PCR; RNA-seq, RNA sequencing; Six1, sine oculis-related homeobox 1; TF, transcription factor. (T [file pbio.3000153.s001.tif]

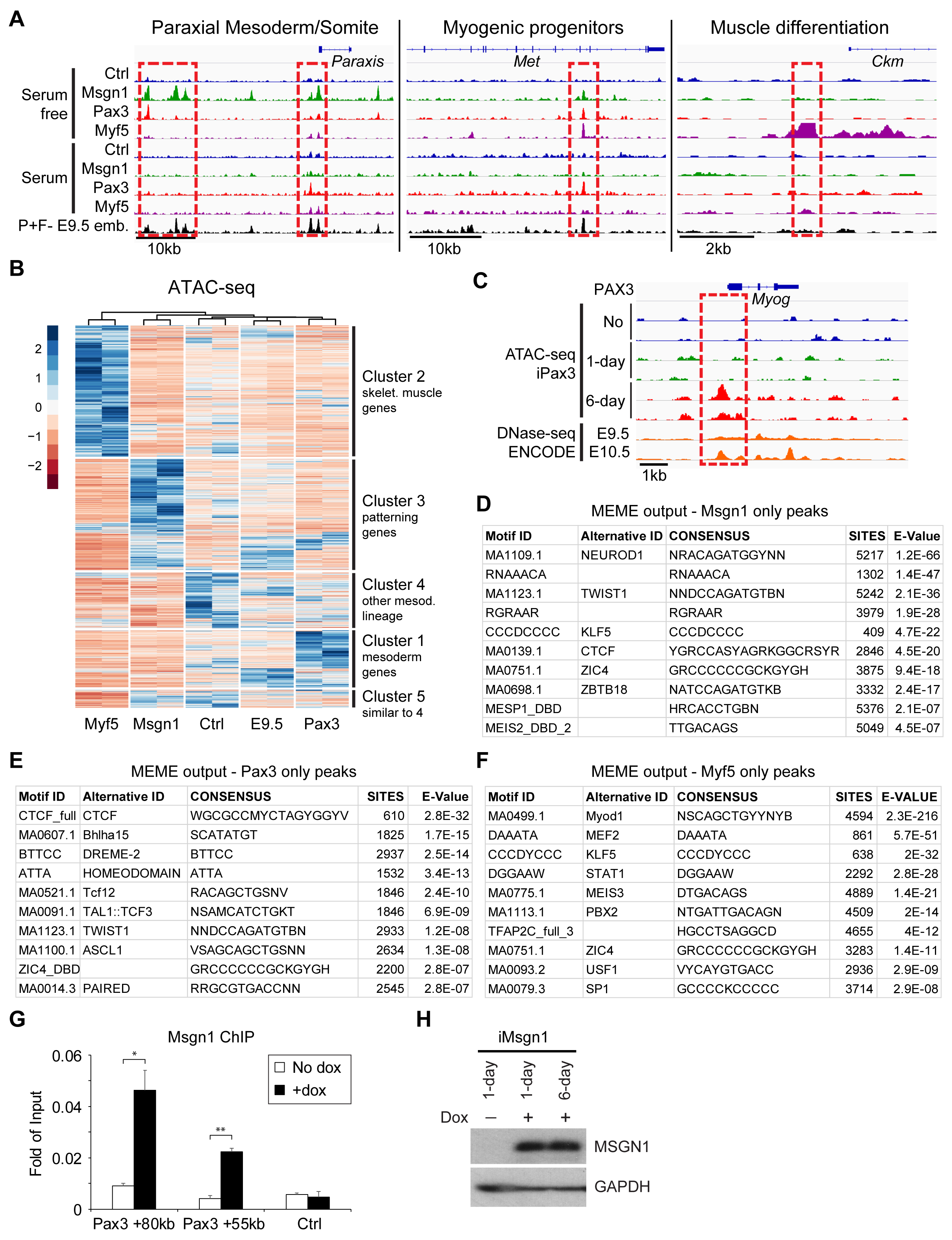

Supplement: S2 Fig — Analysis of ATAC-seq data from iMsgn1, iPax3, and iMyf5 ES cell lines and PDGFRα+FLK1− cells isolated from the trunk region of E9.5 mouse embryos. (A) Representative IGV tracks for genes associated with paraxial mesoderm/somite formation, myogenic progenitor specification, and muscle differentiation and comparison with PDGFRα+FLK1− cells isolated from E9.5 mouse embryos. (B) Heatmap displaying the changes in chromatin accessibility in PDGFRα+FLK1− cells from E9.5 embryos and noninduced, Msgn1-, Pax3-, and Myf5-induced cells from serum-free differentiation. Differential accessible loci from the comparison of each TF versus noninduced cells were combined in a list of unique peaks and used to generate the differential analysis. Five clusters (indicated on the right side) were identified, and the corresponding coordinates were used for GO analysis. Legend indicates the scaled (z score) coverage information for each region. (C) IGV track displaying chromatin accessibility at the Myog locus in cells isolated from 1-day and 6-day Pax3-induced (+) and noninduced (-) EB cultures. Dashed red squares show increased chromatin accessibility at the Myog promoter. This region is a known binding site for muscle regulatory factors. DNase-seq data for E9.5 and E10.5 embryos from Encode consortium are shown below. (D–F) Schematic tables reporting outputs from MEME motif analyses for Msgn1-, Pax3-, and Myf5-induced peaks in serum-free differentiation. (G) ChIP-qPCR validation of Msgn1 binding to the Pax3 locus. Graph represents mean + SD of at least 3 independent biological replicates. *p < 0.05, **p < 0.01. (H) Western blot analysis of MSGN1 expression in Msgn1-induced cultures following 1-day and 6-day doxycycline treatment. GAPDH was used as loading control. Numerical values are available in S1 Data. ATAC-seq, assay for transposase-accessible chromatin sequencing; ChIP, chromatin immunoprecipitation; E, embryonic day; EB, embryoid body; ES, embryonic stem; GAPDH, glyceraldehyde 3-ph [file pbio.3000153.s002.tif]

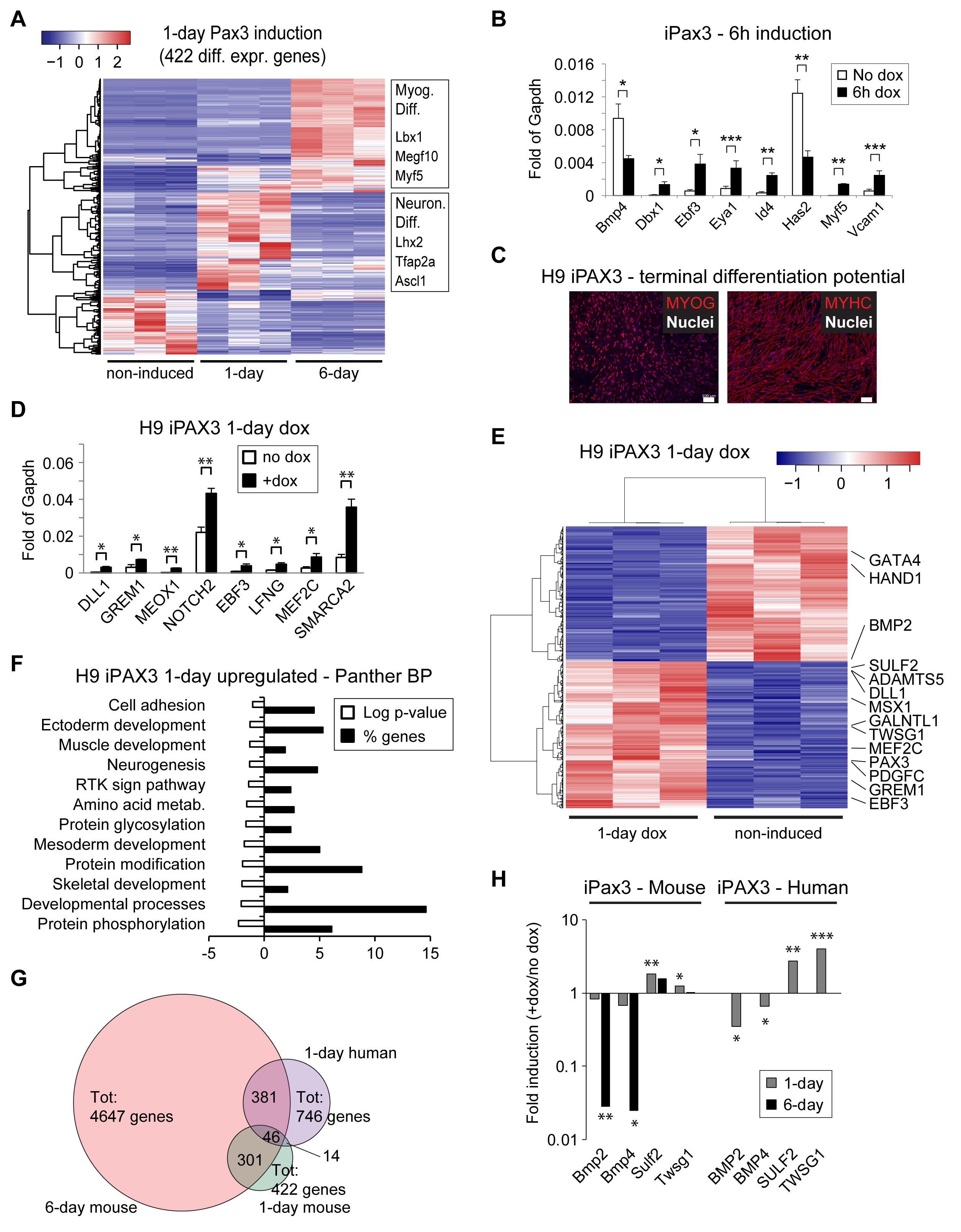

Supplement: S3 Fig — PAX3 transcriptional changes in differentiating human ES cells. (A) Heatmap of genes up-regulated upon 1-day and 6-day Pax3 induction in mouse cells. Changes are relative to noninduced iPax3. A subset of 1-day induced genes is down-regulated in 6-day samples. Selected affected by Pax3 are indicated on the right side of the heatmap. (B) qPCR validation of selected genes from Fig 3. Graph represents mean + SD of at least 3 independent biological replicates. *p < 0.05, **p < 0.01, ***p < 0.001. (C) Immunofluorescence staining for MYOG and MYHC in terminally differentiated cultures from PAX3-induced H9 cells. Left: MYOG (red). Right: MYHC (red). Nuclei (blue). Bar: 100 μm. (D) qPCR analysis of selected genes upon 24 hours of PAX3 expression in differentiating H9 cells. Cells were collected at day 6 of differentiation. Graph represents mean + SD of at least 3 independent biological replicates. *p < 0.05, **p < 0.01. (E) Heatmap of genes up-regulated by PAX3 on day 6 differentiating H9 cells from dox-treated and untreated cultures. (F) Gene ontology analysis of PAX3-up-regulated genes using DAVID. (G) Venn diagram displaying overlap among differentially expressed genes in 1-day and 6-day mouse and 1-day human cells upon Pax3 induction. (H) Gene expression data for Bmp2, Bmp4, Sulf2, and Twsg1 extracted from RNA-seq analysis of Pax3-induced (+dox) and noninduced (no dox) differentiating mouse and human ES cells. Bars represent fold induction (+dox/no dox) of each sample’s mean. *p < 0.05, **p < 0.01, ***p < 0.001. Numerical values are available in S1 Data. Bmp, bone morphogenetic protein; dox, doxycycline; ES, embryonic stem; iPax3, inducible-Pax3; MYHC, myosin heavy chain; MYOG, myogenin; Pax3, paired box 3; qPCR, quantitative PCR; RNA-seq, RNA sequencing; Sulf2, sulfatase 2; Twsg1, twisted gastrulation BMP signaling modulator 1. (TIF) [file pbio.3000153.s003.tif]

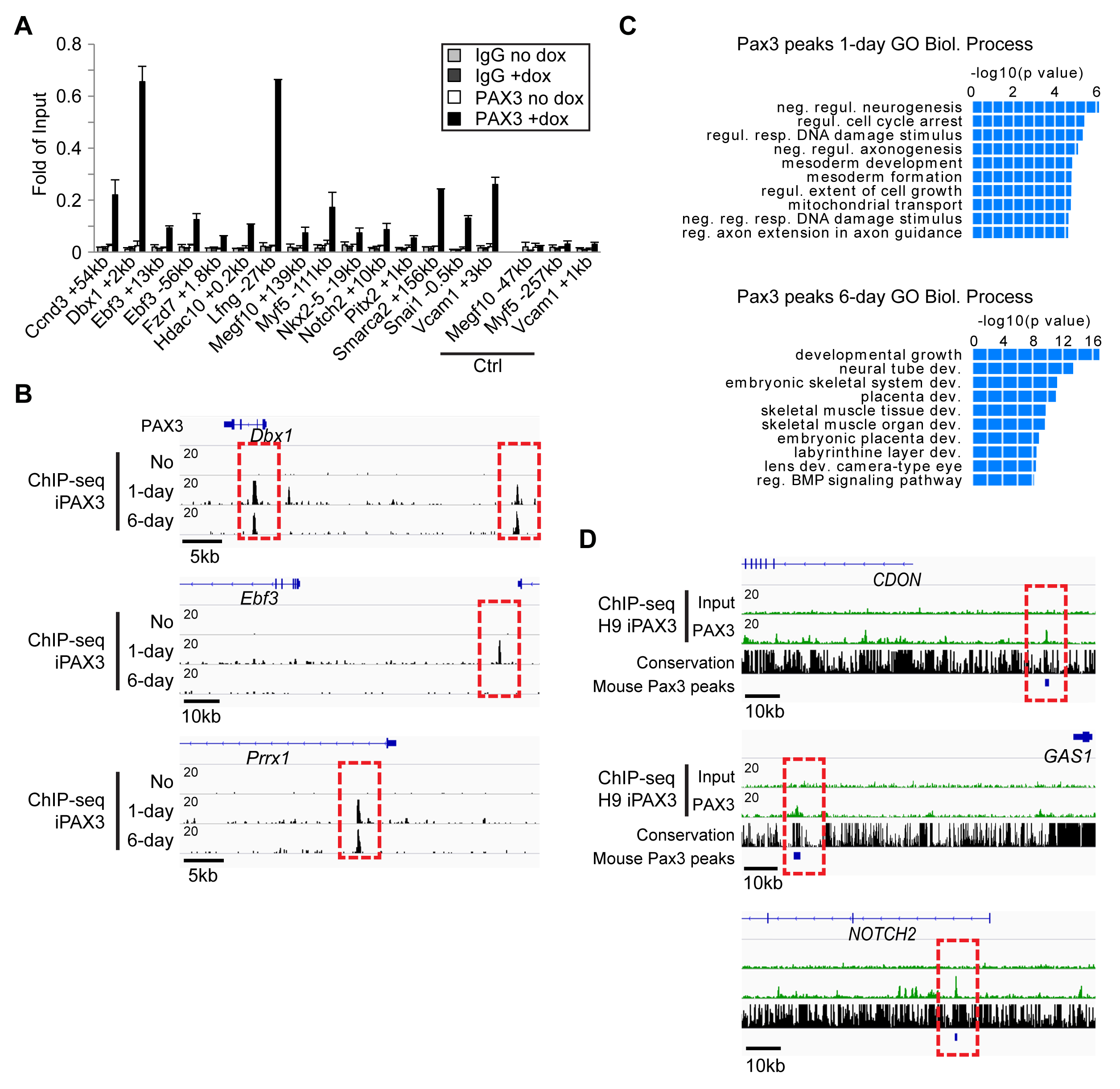

Supplement: S4 Fig — Pax3 genomic occupancy in differentiating mouse and human ES cells. (A) qPCR validation of selected Pax3-bound loci identified by ChIP-seq in day 4 EBs. Mean + SD of at least 3 independent biological replicates is shown. (B) IGV tracks displaying Pax3 genomic occupancy at target gene loci (Dbx1, Ebf3, and Prrx1) in 1-day and 6-day Pax3 ChIP-seq murine datasets. Dashed red squares indicate Pax3 peaks. (C) GREAT functional classification based on biological process of 1-day (3,780) and 6-day (5,710) Pax3 ChIP-seq peaks. Complete annotation data are reported in S2 Table. (D) IGV tracks displaying conserved PAX3 genomic occupancy at loci encoding for Hedgehog (CDON and GAS1) and Notch (NOTCH2) genes in the human genome. Conservation across species and peaks from the 1-day Pax3 ChIP-seq murine dataset (upon genome batch conversion to hg38) are shown below the PAX3 ChIP-seq from differentiating human ES cells. Dashed red squares indicate conserved Pax3 peaks. Numerical values are available in S1 Data. ChIP-seq, chromatin immunoprecipitation sequencing; EB, embryoid body; ES, embryonic stem; IGV, Integrative Genomics Viewer; Pax3, paired box 3; qPCR, quantitative PCR. (TIF) [file pbio.3000153.s004.tif]

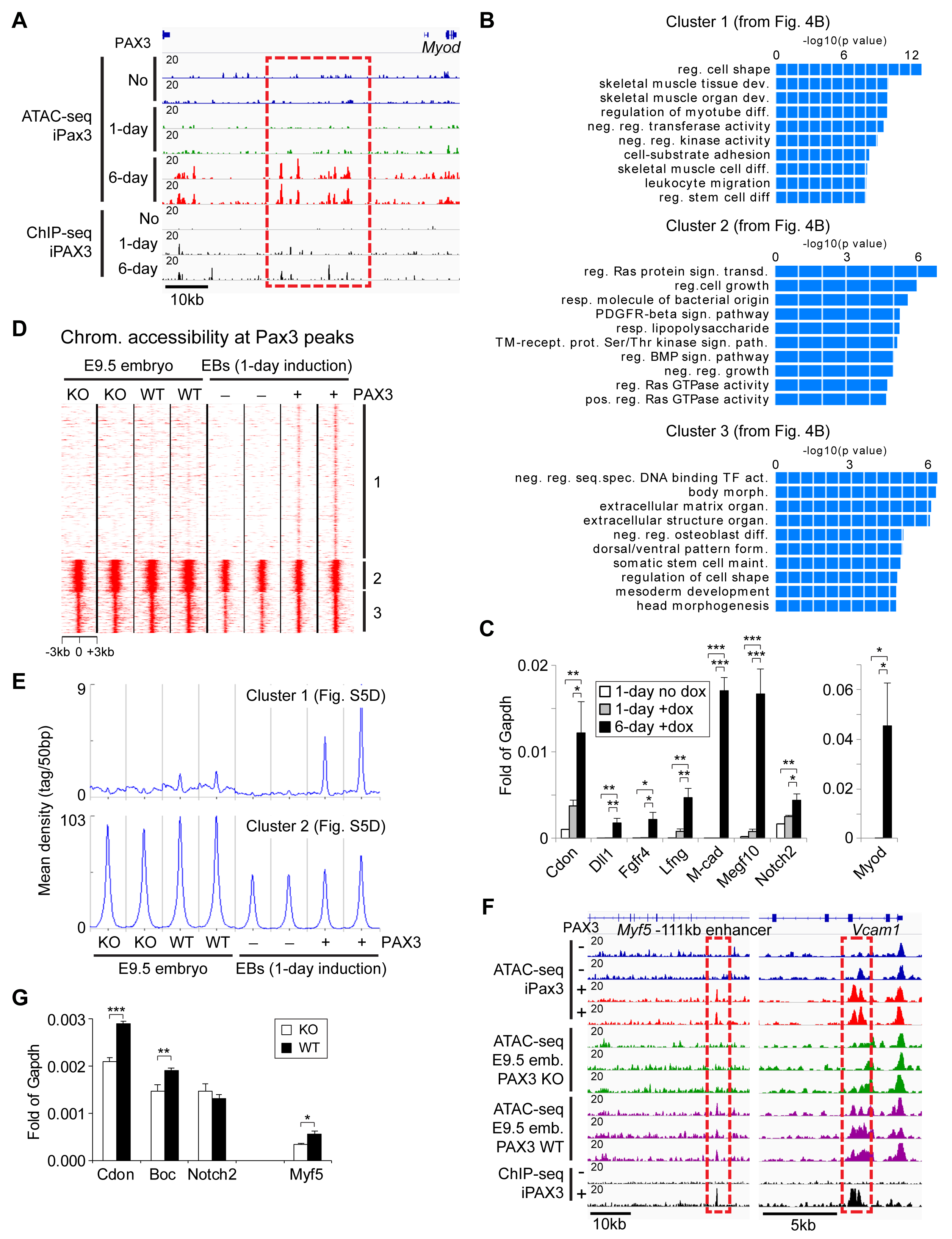

Supplement: S5 Fig — Pax3 regulates chromatin accessibility during myogenic commitment. (A) IGV track displaying chromatin accessibility and Pax3 genomic binding at the Myod locus in cells isolated from 1-day and 6-day Pax3-induced (+) and noninduced (-) EB cultures. Dashed red squares show Pax3-dependent regulation of chromatin accessibility at several Pax3-bound sites. Pax3 binding to these elements are shown by the ChIP-seq tracks. (B) GREAT functional annotation of genomic loci in clusters 1, 2, and 3 from chromatin accessibility analysis of noninduced, 1-day, and 6-day Pax3-induced cells (from Fig 4B). Graphs report biological process classification. Complete annotation data are reported in S2 Table. (C) qRT-PCR analysis of selected genes following 1-day and 6-day Pax3-induction. Mean + SD of at least 3 independent biological replicates is shown. *p < 0.05, **p < 0.01, ***p < 0.001. (D) k-means clustering of ATAC-seq data from PDGFRα+FLK1− cells isolated from Pax3 1-day induced (+) and noninduced (-) day 4 EBs and Pax3 WT and KO E9.5 embryos. Graph represents the ATAC-seq reads overlapping to 1-day Pax3 ChIP-seq peaks (Pax3 peak center ± 3 kb). (E) Distribution of ATAC-seq reads in clusters 1 and 3 from k-means clustering (panel F). (F) IGV track displaying chromatin accessibility at the Vcam1 and Myf5 loci in PDGFRα+FLK1− cells isolated from Pax3-induced (+) and noninduced (-) day 4 EBs and Pax3 WT and KO E9.5 embryos. Dashed red square shows Pax3-dependent regulation of chromatin accessibility at the Pax3-bound Vcam1 +3 kb and Myf5 −111 kb sites. Pax3 binding to Vcam1 +3 kb region and Myf5 −111 kb are shown by the ChIP-seq track. (G) qRT-PCR analysis of selected genes in trunk explants (somite + neural tube) from Pax3-null (KO: n = 4) and WT (n = 3) E9.5 embryos. Graph represents mean + SD of independent biological replicates. *p < 0.05, **p < 0.01, ***p < 0.001. Numerical values are available in S1 Data. ATAC-seq, assay for transposase-accessible chromatin sequencing; ChIP-seq, [file pbio.3000153.s005.tif]

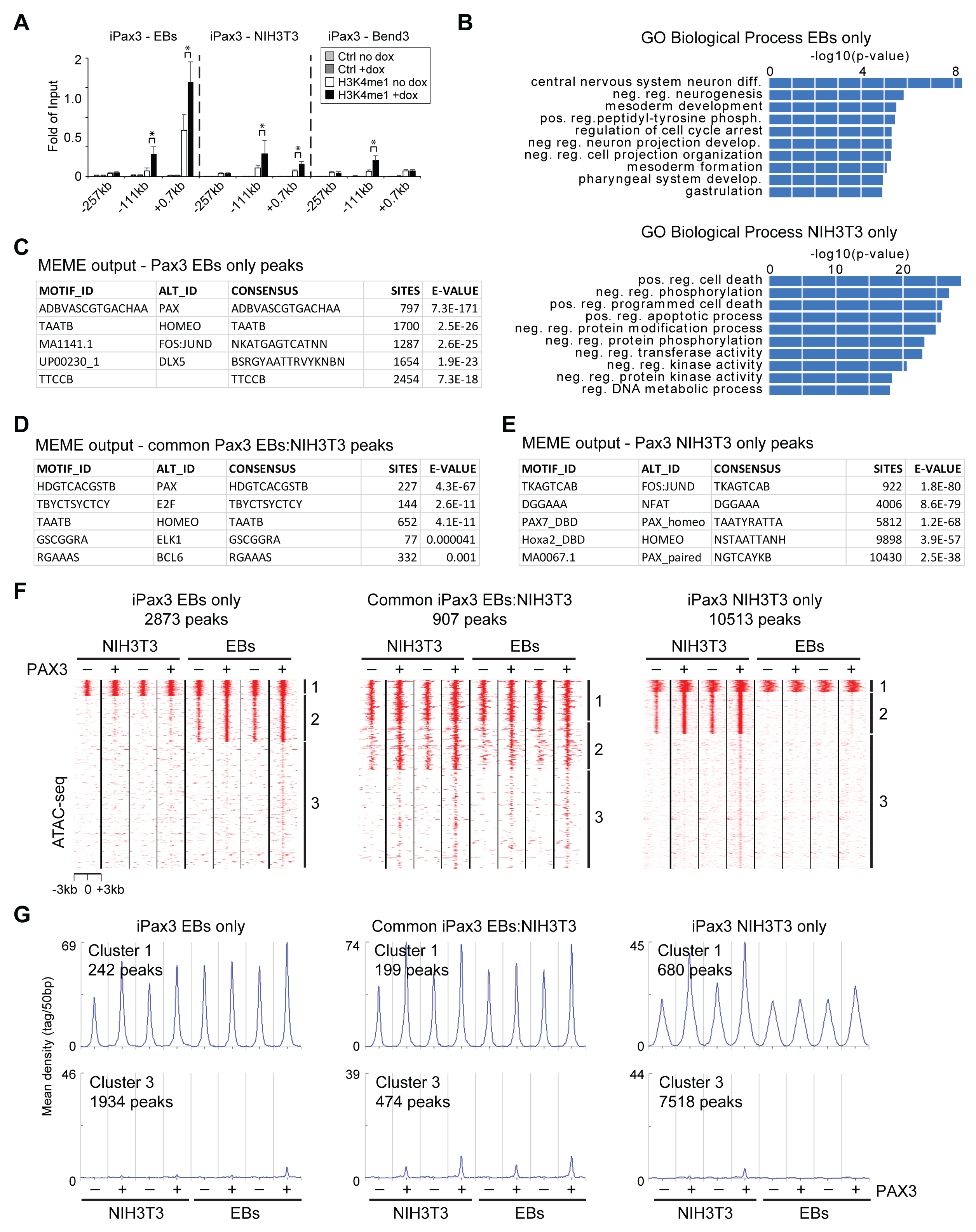

Supplement: S6 Fig — Pax3 does not activate the myogenic program in NIH3T3 fibroblasts and Bend3 endothelial cells. (A) Analysis of H3K4me1 deposition across the Myf5 locus in control (no dox) and Pax3-induced (+dox) day 4 EB, NIH3T3, and Bend3 cells. Graph represents mean + SD from at least 3 independent experiments. *p < 0.05. (B) GREAT functional annotation of peaks from EB-only and NIH3T3-only Pax3 ChIP-seq (from Fig 5D). Graphs report biological process classification. Complete annotation data are reported in S2 Table. (C-E) Schematic tables reporting outputs from MEME motif analyses for EBs-only peaks, common EBs/NIH3T3 peaks, and NIH3T3-only peaks. (F) k-means clustering of ATAC-seq data from 1-day induced (+) and noninduced (-) EB-derived and NIH3T3 iPax3 cells. Graph represents the ATAC-seq reads overlapping to EBs-only, common EBs/NIH3T3, and NIH3T3-only Pax3 ChIP-seq peaks (Pax3 peak center ± 3 kb). (G) Distribution of ATAC-seq reads in clusters 1 and 3 from panel F. Curves show chromatin accessibility centered on EBs-only, common EBs:NIH3T3, and NIH3T3-only Pax3-bound peaks. Datasets are independent biological replicates. Graph represents the ATAC-seq reads overlapping to Pax3 ChIP-seq peaks (Pax3 peak center ± 3 kb). Numerical values are available in S1 Data. ATAC-seq, assay for transposase-accessible chromatin sequencing; ChIP-seq, chromatin immunoprecipitation sequencing; dox, doxycycline; EB, embryoid body; H3K4me1, monomethylated lysine 4 of histone 3; iPax3, inducible-Pax3; Pax3, paired box 3. (TIF) [file pbio.3000153.s006.tif]

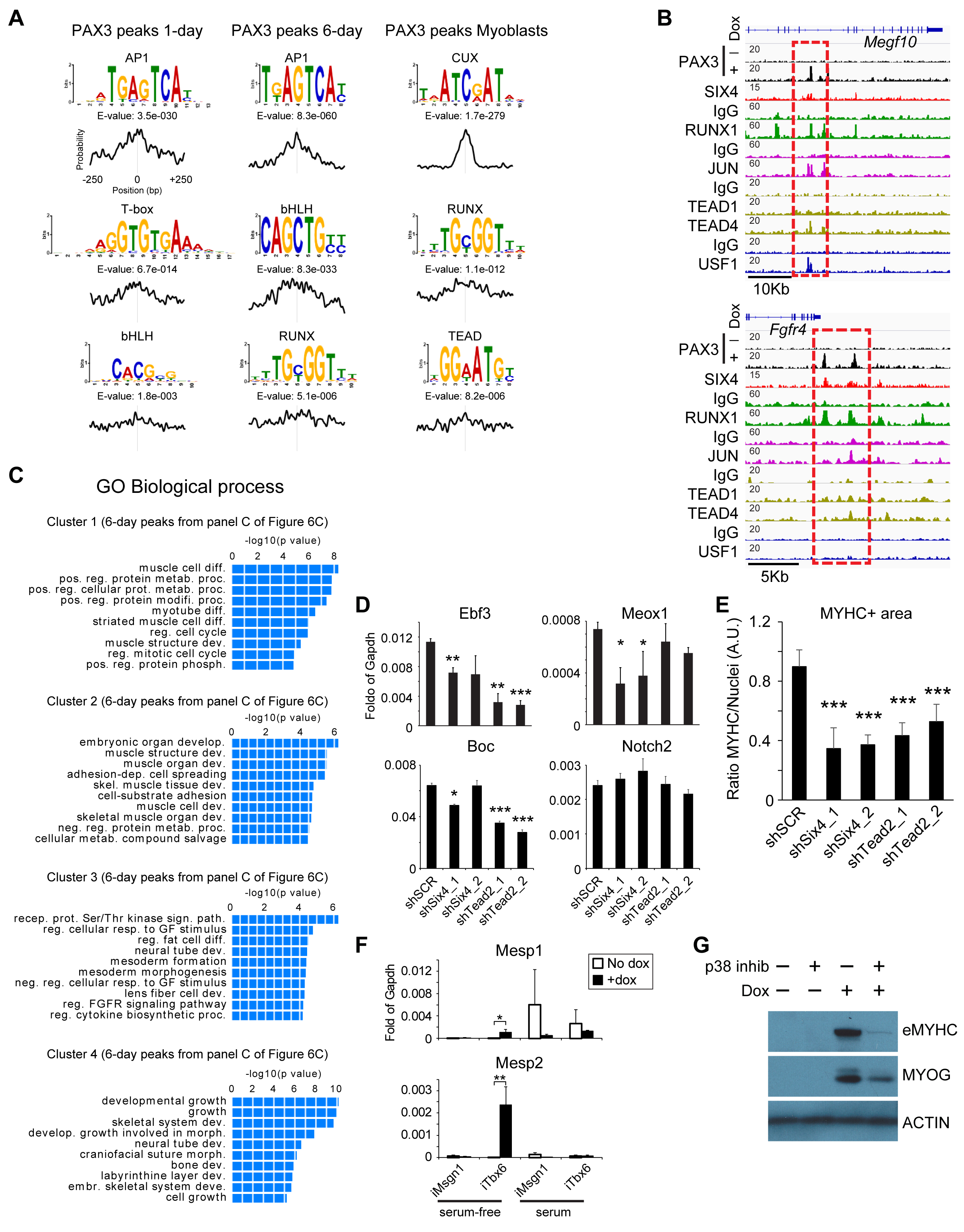

Supplement: S7 Fig — Pax3 cooperates with Six4 and Tead2 to activate the myogenic program. (A) Selected transcription factor motifs enriched at Pax3-bound loci from 1-day, 6-day, and primary myoblasts. Distribution of the motifs across 500 bp from the peak center is reported below. (B) IGV track displaying genomic occupancy for Pax3, Six4, Runx1, Jun, Tead1/4, and Usf1 at the Megf10 and Fgfr4 loci. Dashed red square indicates Pax3-bound site characterized by Six4, Runx1, Jun, and Tead1/4 occupancy. (C) GREAT functional classification of loci from clusters 1, 2, 3, and 4 shown in Fig 6C. Complete annotation data are reported in S2 Table. (D) Gene expression analysis of day 5 EB cells upon Six4 and Tead2 knockdown. Indicated transcripts were analyzed by qRT-PCR with the indicated probes. Graph represents mean + SD from at least 3 independent experiments. (E) Quantification of the MYHC+ area of the immunostaining images shown in Fig 6G. Graph represents mean + SD from at least 3 independent experiments. ***p < 0.001. (F) Expression analysis of Mesp1-2 in nontreated (no dox) and induced (+dox) day 5 EB cells from inducible Msgn1 and Tbx6 ES cell lines differentiated in serum and serum-free conditions. Graph represents mean + SD from at least 3 independent experiments. *p < 0.05, **p < 0.01. (G) Western blot of day 10 cultures from serum differentiation of iPax3 cells. Day 5 EB-derived PDGFRα+FLK1− sorted cells from Pax3-induced (+) and noninduced (-) cultures were treated with the p38 inhibitor SB203580 or the vehicle (DMSO) and collected for analysis after 5 days. eMYHC. MYOG. ACTIN. Numerical values are available in S1 Data. dox, doxycycline; EB, embryoid body; ES, embryonic stem; eMYHC, embryonic MYHC; IGV, Integrative Genomics Viewer; iPax3, inducible-Pax3; Mesp1-2, mesoderm posterior 1–2; Msgn1, mesogenin 1; MYHC, myosin heavy chain; MYOG, myogenin; Pax3, paired box 3; qRT-PCR, quantitative reverse transcription PCR; Runx1, Runt-related transcription factor 1; Tead, TEA domain family m [file pbio.3000153.s007.tif]
